# Modified exosomal SIRPα variants alleviate white matter injury after intracerebral hemorrhage via microglia/macrophages

**DOI:** 10.1186/s40824-022-00311-4

**Published:** 2022-11-26

**Authors:** Xinjie Gao, Heng Yang, Weiping Xiao, Jiabin Su, Yuwen Zhang, He Wang, Wei Ni, Yuxiang Gu

**Affiliations:** 1 Department of Neurosurgery, Huashan Hospital, Fudan University, Shanghai, 200040 China; 2 Shanghai Key Laboratory of Brain Function and Restoration and Neural Regeneration, Shanghai, 200052 China; 3 Neurosurgical Institute of Fudan University, Shanghai, 201107 China; 4 Institute of Science and Technology for Brain-Inspired Intelligence, Fudan University, Shanghai, 200433 China; 5 National Center for Neurological Disorders, Shanghai, 201107 China

**Keywords:** Exosome, SIRPα variant, Intracerebral hemorrhage, White matter injury, Microglia/macrophages

## Abstract

**Background:**

Despite limited efficiency, modulation of microglia/macrophages has shown to attenuate neuroinflammation after intracerebral hemorrhage (ICH). In this context, we evaluated the efficacy of modified exosomal signal regulatory protein α (SIRPα) variants (SIRPα-v Exos) in microglia/macrophages and neuroinflammation-associated white matter injury after ICH.

**Methods:**

SIRPα-v Exos were engineered to block CD47-SIRPα interactions. After obtaining SIRPα-v Exos from lentivirus-infected mesenchymal stem cells, C57BL/6 mice suffering from ICH underwent consecutive intravenous injections of SIRPα-v Exos (6 mg/kg) for 14 days. Afterwards, the volume of hematoma and neurological dysfunctions were assessed in mice continuously until 35 days after ICH. In addition, demyelination, electrophysiology and neuroinflammation were evaluated. Furthermore, the mechanisms of microglial regulation by SIRPα-v Exos were investigated in vitro under coculture conditions.

**Results:**

The results demonstrated that the clearance of hematoma in mice suffering from ICH was accelerated after SIRPα-v Exo treatment. SIRPα-v Exos improved long-term neurological dysfunction by ameliorating white matter injury. In addition, SIRPα-v Exos recruited regulatory T cells (Tregs) to promote M2 polarization of microglia/macrophages in the peri-hematoma tissue. In vitro experiments further showed that SIRPα-v Exos regulated primary microglia in a direct and indirect manner in synergy with Tregs.

**Conclusion:**

Our studies revealed that SIRPα-v Exos could accelerate the clearance of hematoma and ameliorate secondary white matter injury after ICH through regulation of microglia/macrophages. SIRPα-v Exos may become a promising treatment for ICH in clinical practice.

**Graphical Abstract:**

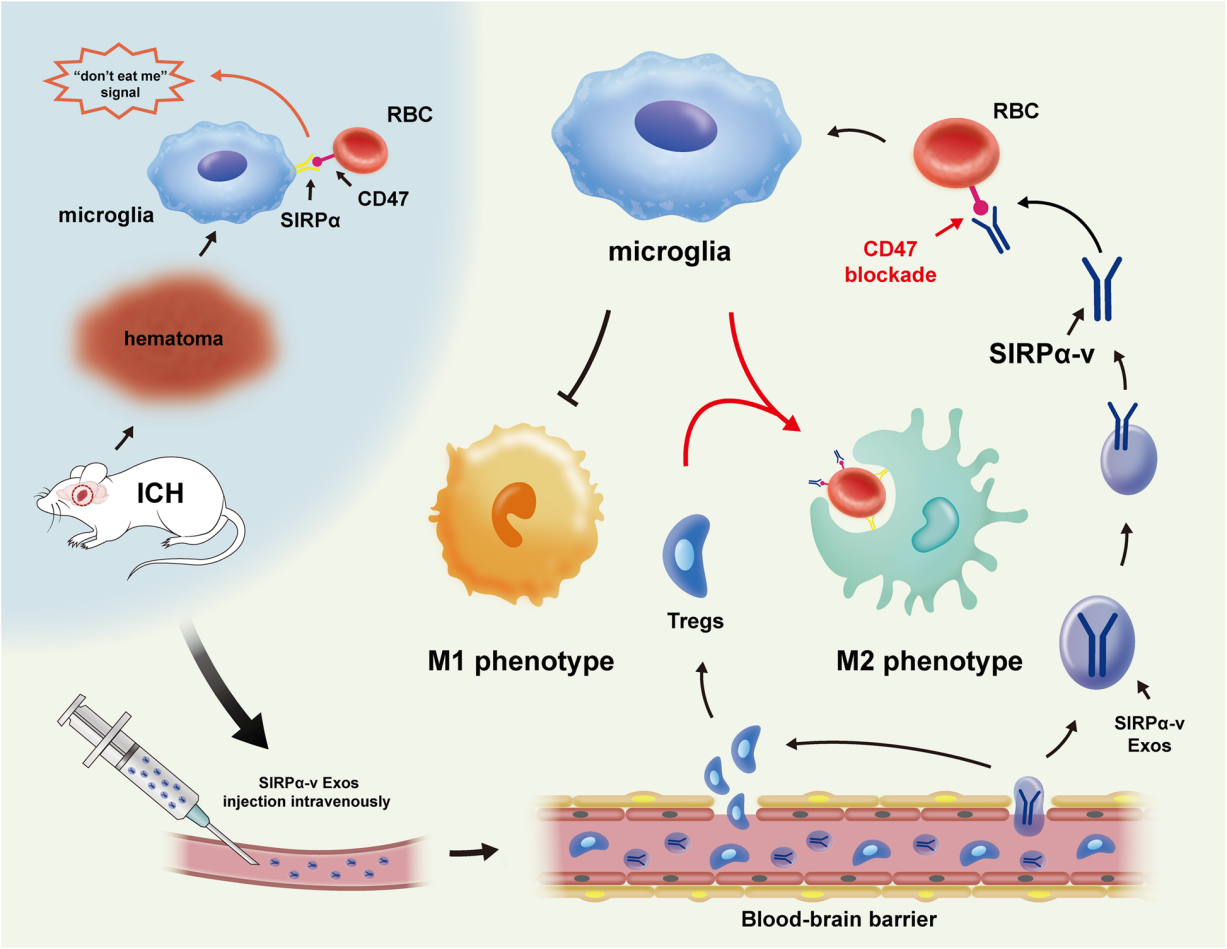

**Supplementary Information:**

The online version contains supplementary material available at 10.1186/s40824-022-00311-4.

## Introduction

Intracerebral hemorrhage (ICH) is the second most common subtype of stroke, which has become an important global public health problem [1]. The mortality of ICH is more than 30% in the acute phase, and 70 ~ 80% of patients survive with neurological dysfunction [2]. The rapid formation of hematoma in the brain parenchyma disrupts the brain tissue. The nerve bundles surrounding the hematoma are displaced and deformed due to the occupancy. Owing to the lower toxicity of fresh blood, extensive edema is rarely observed in the early stage of ICH. Intracerebral injection of intact erythrocytes does not incur considerable edema within 24 h, whereas injection of lysed erythrocytes or hemoglobin does [3]. The products of lysed erythrocytes in hematoma subsequently induce neuroinflammation, leading to secondary brain injury. Plasma precipitation further aggravates neuroinflammation. Besides, the release of vasoactive substances from lysed erythrocytes causes cerebral vasospasm around the hematoma. All of the above factors contribute to severe secondary brain injuries in patients with hematoma that has not been promptly removed. However, regarding intracerebral hematoma, apart from surgical evacuation, few effective options can be adopted.

White matter injury (WMI) is considered one of the main factors of long-term neurological impairment after ICH [4, 5]. Disruption of white matter is evident on magnetic resonance imaging (MRI) in patients with ICH, associating with poor prognosis [6]. Disintegration of axons and myelin sheaths could be exacerbated by subsequent neuroinflammation [7]. Clearance of erythrocytes prior to their lysis may assuage secondary WMI from subsequent neuroinflammation [3, 8].

Regarded as resident macrophages in the central nervous system (CNS), microglia play a critical role in neuroinflammation. Generally, in CNS diseases, microglia can polarize into M1- and M2-phenotypes when stimulated by different factors [9, 10]. The M1-phenotype secretes toxic reactive oxygen intermediates and inflammatory cytokines, while the M2-phenotype is responsible for pathogen removal as well as neural regeneration and repair [11]. Different phenotypes of microglia/macrophages play opposite roles in ICH. The M1-phenotype has been shown to exacerbate neuronal demise through a pro-inflammatory response. In contrast, the M2-phenotype is related to scavenging cell debris and secreting protective cytokines, which are essential for brain recovery [12, 13]. Given the dual role of microglia/macrophages in CNS diseases, pharmacologically and genetically manipulating the differentiation of microglia/macrophages into the M2-phenotype rather than the M1-phenotype has been proven to facilitate the clearance of hematoma and alleviate inflammatory responses [12, 14, 15]. Accumulating evidence has shown that regulating microglia/macrophages via the CD47-SIRPα pathway changes the phagocytic capacity. As a typical inhibitory immune receptor, SIRPα is selectively expressed on myeloid cells, including microglia, macrophages, and granulocytes [16–18]. Abundantly expressed on the membrane of erythrocytes [15], CD47 has been identified as an antiphagocytic marker [19] and activates a “do not eat me” signal through binding to SIRPα, leading to the inhibition of phagocytosis of erythrocytes [20, 21]. Macrophages are robustly stimulated when CD47 is blocked. Antibodies that block CD47 binding to SIRPα have been reported to increase the phagocytosis of target cells [22, 23]. However, it has been documented that antibodies do not achieve their maximal efficacy when CD47 is free to transduce inhibitory signals through SIRPα on macrophages [24]. Hence, we sought to design a new substance that might efficaciously block the interaction of CD47 and SIRPα, enhancing the phagocytic capacity of microglia/macrophages.

With biological properties of stemness and pluripotency, mesenchymal stem cells (MSCs) have been widely explored in fundamental and preclinical research in terms of regeneration and differentiation [25, 26]. Exosomes secreted by MSCs, as naturally secreted lipid bilayer membrane-enclosed vesicles, exert neuroprotective effects in many CNS diseases [27–29]. Exosomes are able to cross the blood–brain barrier (BBB) and deliver their payload to receptor cells with virus-like efficiency [30]. Drugs modified by exosomes could contribute to their application in CNS diseases. The miRNA-17–92 encapsuled by MSC-exosomes promoted oligodendrocyte regeneration, increased neurological plasticity, and preserved neurological functions after stroke in rats [31]. Modified exosomal rabies virus glycoprotein derived from MSCs exhibited preferable hippocampal targeting and rescued memory deficits by regulating inflammatory responses in Alzheimer’s disease [32].In addition, MSC-exosomes attenuate the inflammatory response and offer neuroprotection and functional improvements by reducing neuronal apoptosis in models of traumatic brain injury and ICH [33, 34].

Thus, we designed modified exosomes consisting of two characteristics. On the one hand, exosomes act as nanocarriers for delivery, efficiently penetrating the BBB. On the other hand, high-affinity SIRPα variants, as CD47 antagonists, were engineered to interfere with CD47-SIRPα signaling between erythrocytes and microglia/macrophages. In this study, we used in vivo and in vitro preclinical models to delineate the effects and mechanisms of modified exosomal SIRPα variants. We show that modified exosomal SIRPα variants block the CD47-SIRPα interaction and promote hematoma clearance, relieving WMI after ICH. Our data provide a potential therapeutic strategy and lay a foundation for further insight into the pathophysiological mechanism of ICH.

## Materials and methods

### Animals

C57BL/6 mice were purchased from the Slac Laboratory Animals Center (Shanghai, China). All experimental protocols were approved by the Department of Laboratory Animal Science, Fudan University (Shanghai, China). Mice were given ad libitum access to food and water.

### Expression and purification of SIRPα variants

Accordingly [35], the sequence encoding NH_2_-NdeI-GST-SIRPa variant-Hind III-COOH was ligated to the pET-28a ( +) plasmid vector to obtain SIRPα variants. Then, the plasmid was transferred into BL-21(DE3) *E. coli* to express a GST-fusion SIRPα variant with a histidine tag. The transformed *E. coli* cells were incubated with shaking in LB medium containing kanamycin at 37 °C for 24 h. When the OD_600_ reached 0.8, cells were induced with 1 mM isopropyl-β-D-thiogalactoside (IPTG) to express SIRPα variants. GST-fusion SIRPα variants were purified by nickel-nitrilotriacetic acid (Ni–NTA) chromatography and identified with anti-GST antibody. Eluted GST-fusion SIRPα variants were subsequently digested with HRV 3C protease at 4 °C for 12 h to remove the GST tag. To obtain biotinylated CD47 and SIRPα WT/variants, proteins were expressed with an Avi tag and purified as described above. After biotinylated by BirA ligase in vitro, the biotinylated proteins were repurified through size-exclusion chromatography from the mixture.

### Surface plasmon resonance (SPR)

After quantifying the protein concentrations with a xMark spectrometer (Bio-Rad Laboratories Inc., Hercules, CA, USA), biotinylated CD47 (R_max_ ~ 150 RU) was captured with Biacore SA sensor chips (GE Health, Boston, MA, USA) at 25 °C. Unrelated biotinylated proteins were applied to control nonspecific binding. The response of SIRPα variants was measured with serial dilutions in HBS-P buffer (GE Healthcare). The CD47 surface was regenerated by three 60-s injections of 2 mol/L MgCl_2_. All data were analyzed with Biacore T200 (GE Healthcare) evaluation software with the Langmuir binding model.

### In vitro CD47 binding and blocking analyses

As reported previously [24], 1 × 10^6^ erythrocytes were incubated with biotinylated SIRPα WT/variants or anti-CD47 antibody for 30 min at 4 °C for cell binding analysis. The binding of biotinylated proteins was detected by Alexa Fluor 594-conjugated streptavidin as a secondary staining reagent. In cell blocking analysis, biotinylated SIRPα WT was incubated with Alexa Fluor 594-conjugated streptavidin to form SIRPα WT tetramers. Then, SIRPα WT tetramers were combined with CD47 antagonists added to CFSE^+^ erythrocytes simultaneously. After removal of the remaining nonspecific signals, the cells were analyzed by flow cytometry. Data for the above experiments represented the fluorescence intensity normalized to maximal binding and were fitted to a dose–response curve.

### Isolation and cultivation of MSCs

Primary mouse MSCs were isolated from the bone marrow of wild-type 8-week-old C57BL/6 mice. In brief, the femur and tibia were dissected from mice, and bone marrow was flushed out with complete isolation media (CIM) consisting of Dulbecco’s modified Eagle’s medium (DMEM) supplemented with 10% fetal bovine serum (FBS; Thermo Fisher Scientific, Waltham, MA, USA), 100 U/mL penicillin (Thermo Fisher Scientific), and 100 μg/mL streptomycin (Thermo Fisher Scientific). Cells were washed with phosphate-buffered saline (PBS), filtered through a 70-μM nylon mesh filter, and cultured in 40 mL CIM. Cells were passaged every 1 week using 0.25% trypsin/1 mM ethylenediaminetetraacetic acid (EDTA).

### Lentivirus preparation and gene delivery in vitro

The sequence of the final type SIRPα variant was inserted into the preprepared pCDH1-MSCV-MCS1-EF1-GreenPuro vector. After plasmid recombination was completed, the plasmid was amplified and transfected into 293 T cells to coat the lentiviral vector. The lentivirus containing the SIRPα variant sequence was directly added to the medium, and MSCs were then transfected at a multiplicity of infection (MOI) of 5. The same amount of vector (NC Exo group) was also added to the medium as an experimental control. Then, the cells were incubated at 37 °C in a 5% CO_2_ incubator for another 3 days or at 80–90% confluency in a 10-cm dish before exosome extraction.

### Isolation and identification of exosomes

After transfection, the medium was changed to serum-free DMEM/F12 medium and maintained in 5% CO_2_ at 37 °C. To isolate exosomes, the supernatants were harvested 72 h after replacement with serum-free medium and then subjected to differential centrifugation. Briefly, the medium containing exosomes was centrifuged at 300 × g for 10 min, 2,000 × g for 10 min, and 10,000 × g for 30 min to remove cellular debris and other cellular components. components. After filtering with a 0.22 μm pore filter, the medium was ultracentrifuged at 36,900 rpm for 2 h in a 70 Ti rotor (Beckman Instruments). The obtained exosomes were resuspended in PBS containing protease inhibitors.

### Transmission electron microscopy (TEM)

TEM was performed as described previously [36]. Briefly, mice were perfused with saline, followed by ice-cold 4% paraformaldehyde and 0.1% glutaraldehyde in 0.1 mol/L PBS (pH 7.4). The peri-hematoma area tissue was micro-dissected into 1 mm blocks and fixed in 2% glutaraldehyde overnight. Next, tissues were washed in 0.1 mol/L sodium cacodylate buffer (pH 7.4) and postfixed in buffered osmium tetroxide for 1 ~ 2 h. Following serial dehydration in acetone, the tissue was embedded in epoxy resin and sectioned to 60 ~ 90 nm thickness. The exosome samples or brain tissue were placed onto 200 mesh grids, stained with uranyl acetate and lead citrate, and examined with a JEOL JEM-1230 transmission electron microscope (JEOL Ltd., Tokyo, Japan).

#### Exosome biodistribution

Briefly [37], 1 mg of modified exosomal SIRPα variants (namely SIRPα-v Exo) was labeled with 10 μg of DiIC18(7) (1,1’-dioctadecyl-3,3,3’,3’-tetramethylindotricarbocyanine iodide) (DiR) (Thermo Fisher Scientific) to obtain DiR-labeled SIRPα-v Exos. Ten milligrams/kg DiR-labeled SIRPα-v Exos were injected through the tail vein (volume 200 μL). For noninvasive imaging of SIRPα-v Exo biodistribution, mice were anesthetized (2–3% isoflurane, 100% oxygen) and placed in the VISQUE™ Imaging System (Vieworks, Inc., Gyeonggi-do, Republic of Korea). A 750 nm channel was used to excite the DiR and fluorescence signal was observed. Twenty-four hours after injection, the animals were euthanized, and their livers, kidneys, spleens, lungs, hearts and brains were collected. These organs were weighed and imaged under the same imaging system and the amount of DiR dye was quantified by CleVue™ software.

### Establishment of a mouse model of ICH

Intracerebral injections were performed as previously described [15]. Briefly, mice were anesthetized with a 3% isoflurane/air mixture until they were unresponsive in the tail-pinch test. Body temperature was maintained at 37.5 °C by a feedback-controlled heating pad. Then, the mice were positioned in a stereotaxic frame, and a cranial burr hole (1 mm) was drilled near the right coronal suture. A 26-gauge needle was inserted stereotaxically into the right basal ganglia (coordinates: 0.2 mm anterior, 3.5 mm ventral, and 2.5 mm lateral to the bregma). Either 30 µL autologous whole blood was infused at 2 µL/min by a microinfusion pump. After injection, the needle remained in the position for 10 min to prevent reflux, and then, it was gently removed. The burr hole was filled with bone wax, and the skin incision was closed. Mice suffering from ICH underwent intravenous injection of exosomes for 14 consecutive days at a concentration of 6 mg/kg. Mice in the sham-operated group underwent the same anesthesia and exposure of the brain without injection.

### 11.7 T magnetic resonance imaging (MRI)

Mice were anesthetized with a 3% isoflurane/air mixture throughout the 11.7 T MRI scan. The 11.7 T MRI was performed on the 1st, 3rd, 7th and 35th days post-ICH by an 11.7 T MRI system (Bruker BioSpec, Karlsruhe, Germany) at the Institute of Science and Technology and Brain-inspired Intelligence (Shanghai, China). The imaging protocol for all the mice included T2 fast spin-echo (repetition time/echo time = 4000/60 ms) and SWI sequences (repetition time/echo time = 250/5 ms). The field of view was 20 × 20 mm, and the matrix was 256 × 256 mm. Twenty-five coronal slices (thickness, 0.5 mm) were acquired from the frontal pole to the brain stem. Afterwards, the SWI lesion volume was calculated by ImageJ software (National Institutes of Health, Bethesda, MD, USA). The SWI lesion was outlined along the border of the hypointense area, and the lesion volume was obtained by combining the hypointense area over all slices. The DTI sequence was only performed on the 35th day post-ICH.

### Morris water maze test

The Morris water maze test was performed to assess spatial memory on the 35th day post-ICH. All tests were performed by researchers who were blinded to the experiments. Mice were trained to find an escape platform (11 cm diameter). The hidden platform test assessed the ability of mice to find the platform without being able to observe it directly, and mice had to remember the location of the platform relative to external spatial cues. The platform was placed 1 cm under the water surface, and water was made opaque with white, nontoxic tempera paint. Each mouse was released from one of four locations and was allotted 90 s to search for the hidden platform. At the end of each trial, the mouse was placed on the platform or was allowed to remain on the platform for 30 s with prominent spatial cues displayed around the room. Four trials were undertaken per day for five consecutive days, and the location of the platform was kept constant. Data are expressed as time (in seconds) or latency to reach the submerged platform each day. After the last day of the hidden platform test, a single 60-s probe trial was conducted. The platform was removed, and each mouse was placed in pool for 60 s at the same initial location that was used during the primary hidden platform test. Time spent in the goal quadrant (where the platform had been located) and swimming speed were both recorded.

### Behavioral tests

Behavioral tests were conducted by a researcher who was blinded to the experiments. Sensorimotor functions were measured at 1 ~ 35 days after ICH. Before performing the behavioral tests, all animals were subjected to behavior training for three days, and animals displaying abnormal behavior were excluded.

#### Foot-fault test

Foot-fault test was undertaken to evaluate dysfunction of the forelimbs and hind limbs. Mice were placed on an elevated grid surface (30 (L) × 35 (W) × 31(H) cm^3^) with a grid opening of 2.5 cm^2^. Each fall and slip between the wires with weight-bearing steps could be recorded as a forelimb or hindlimb foot-fault. The calculation formula of the paw fault ratio is as follows:

$$\text{paw fault ratio}=\frac{\text{paw fault s}\text{teps}}{\text{total steps}}\times\;100\%$$

#### Adhesive removal test

Adhesive removal test was performed to assess tactile responses and sensorimotor asymmetries. Two 2 × 3-mm adhesive tapes were applied to lesioned forepaws. Tactile responses were measured by recording the time of initial contact with ipsilateral paws, as well as the time to remove the adhesive tape, with a maximum observation period of 120 s.

### Measurement of compound action potentials (CAPs)

CAPs in the CC were measured as described previously [36]. The brain tissue was cut into coronal slices and placed in pregassed (95% O_2_/5% CO_2_) artificial cerebrospinal fluid (aCSF: 126 mmol/L NaCl, 2.5 mmol/L KCl, 1 mmol/L Na_2_H_2_PO_4_, 2.5 mmol/L CaCl_2_, 26 mmol/L NaHCO_3_, 1.3 mmol/L MgCl_2_, and 10 mol/L glucose; pH 7.4) for 1 h at room temperature. Slices were perfused with aCSF at a constant rate (3 ~ 4 mL/min) at 22 °C. A bipolar tungsten-stimulating electrode was positioned across the CC 0.9 mm lateral to the midline. A glass extracellular recording pipette was placed in the external capsule. Only recordings at 0.48 mm from the stimulating electrode were reported in the current study. Both electrodes were placed 50 ~ 100 mm below the surface of the slice, with adjustments to optimize the signal. The CAP was amplified (× 1 k) and recorded using an Axoclamp 700B (Molecular Devices, Inc., San Jose, CA, USA) and then analyzed using pCLAMP 10.0 software (Molecular Devices, Inc.). Input–output curves were generated by varying the intensity of the stimuli from 0.25 to 2 mA in 0.25-mA increments. The average waveforms of four successive sweeps in two slices per animal were analyzed. The amplitude of the N1 component of the CAP (representing myelinated fibers) was quantified as the difference from the first positive peak to the first negative peak.

### Immunohistochemistry, cell counting, and fluorescence quantification

Mice were euthanized with sodium pentobarbital and perfused with 4% paraformaldehyde in 0.1 mM PBS (pH 7.4). Brains were harvested and cryoprotected in 30% (wt/vol) sucrose solution for 24 h. The brain tissues were then frozen with OCT compound (Sakura Finetek, Inc., Torrance, CA, USA). The sections were blocked with 10% (vol/vol) normal donkey serum for 1 h, followed by overnight incubation (4 °C) with the following primary antibodies: rat anti-MBP (Abcam, Cambridge, UK), mouse anti-SMI32 (BioLegend, San Diego, CA, USA), rabbit anti-Iba1 (Wako, Tokyo, Japan), goat anti-CD206 (Abcam), and rat anti-CD16/32 (Abcam). All the primary antibodies used are listed in Table S2. The appropriate Alexa Fluor-conjugated antibodies (Jackson ImmunoResearch Laboratories, Inc., West Grove, PA, USA) were used as secondary antibodies. Finally, the fluoroshield with DAPI (Sigma-Aldrich, St. Louis, MO, USA) was used to mount the cover slides. Cell numbers were calculated per square millimeter from 2 random microscopic fields on 4 sections (a total of eight images) cut through the STR. All procedures were performed in a blinded fashion. The fluorescence intensity of MBP and SMI32 was measured to demonstrate axonal damage after ICH.

### Quantitative reverse transcription polymerase chain reaction (RT-qPCR)

Briefly, total RNA was extracted from the ipsilateral striatum on the 1st, 3rd, 7th, and 14th days post-ICH or from sham-operated mice using TRIzol reagent (Thermo Fisher Scientific). RNA was reversely transcribed into cDNA using the Superscript First-Strand Synthesis System (Invitrogen, Carlsbad, CA, USA). RT-qPCR was performed using the Opticon 2 Real-Time PCR Detection System (Bio-Rad Laboratories Inc.) and SYBR gene PCR Master Mix (Invitrogen). Cycle threshold values were measured through the auto Ct function. The sequences of the primers used for RT-qPCR are listed in Table S3.

### Flow cytometry

Animals were euthanized and perfused with cold saline. Brains were dissected, and the ipsilateral (right) and contralateral (left) hemispheres were collected. Brains were dissociated into a single-cell suspension using a gentle magnetic-activated cell sorting (MACS) Dissociator (Miltenyi Biotec, Bergisch Gladbach, Germany) following the manufacturer’s instructions. The suspension was passed through a 70-μm cell strainer (Millipore, Bedford, MA, USA) and resuspended in Percoll (GE Health, Boston, MA, USA). Single-cell suspensions were separated from myelin and debris by centrifugation (500 g, 30 min, 18 °C) on a 30 ~ 70% Percoll gradient. Cells at the interface were collected and washed with Hank’s balanced salt solution (HBSS; Sigma-Aldrich) containing 1% FBS (Sigma-Aldrich) and 2 mM EDTA (Sigma-Aldrich) before staining. Single-cell samples were first incubated with antibodies against surface antigens for 30 min on ice at 4 °C in the dark. After washing twice, the cells were fixed and permeabilized with an intracellular staining kit (Thermo Fisher Scientific) according to the manufacturer’s protocol. The antibodies used were as follows: anti-CD29-APC (eBioscience, Waltham, MA, USA), anti-CD44-FITC (Abcam), anti-CD90-FITC (Abcam), anti-CD11b-APC (Cell Signaling Technology, Inc., Danvers, MA, USA), anti-CD34-PE (Abcam), anti-CD45-PE (Cell Signaling Technology), anti-CD4-APC (Abcam), and anti-CD25-FITC (Abcam). Appropriate isotype controls were used according to the manufacturer’s instructions (Thermo Fisher Scientific). Fluorochrome compensation was performed with single-stained OneComp eBeads (Thermo Fisher Scientific). Flow cytometry was undertaken using a BD LSRII flow cytometer (BD Biosciences, Franklin Lakes, NJ, USA). Data analyses were carried out by FlowJo 10.0 software. In addition, 1 × 10^6^ cells were collected from each sample.

### Isolation and culture of primary microglia

Primary microglia were collected as described previously [36]. Mouse primary microglia were isolated from neonatal C57BL/6 mice. Briefly, the forebrains were completely digested with 0.125% trypsin (Thermo Fisher Scientific) and DNase (Sigma-Aldrich) and centrifuged at 1500 rpm for 15 min. The cell pellet was suspended in culture medium and filtered with a 40-μm cell filter (Millipore). Then, the cell mixture was incubated in a poly-D-lysine pre-coated flask for 10 days. Microglia were detached after shaking the flask at 220 rpm for 1 h at 37 °C. The supernatant containing microglia was collected and centrifuged at 1000 rpm for 5 min. Then, microglia were routinely cultured in DMEM/F12 (Thermo Fisher Scientific) with 10% FBS.

### Isolation and culture of Tregs

Briefly, C57BL/6 mice were sacrificed to collect spleens, which were ground and blown into a celiac-like mixture. The mixture was filtered through a 70-µm cell sieve. The single-cell suspension was treated with 2 mL of erythrocyte lysis solution for 10 min, followed by 10 mL of complete medium to terminate the lysis. The cells were centrifuged and washed 3 times before sorting. RPMI-1640 medium (Thermo Fisher Scientific) was used to resuspend the cells. A MACS kit (Miltenyi) was applied to sort CD4 CD25-positive T cells. Then, the cells were eluted and collected from the magnetic beads with RPMI-1640 medium. After centrifugation, Tregs were resuspended in fresh RPMI-1640 medium and cultured in a 37 °C incubator.

### In vitro phagocytosis assay

Erythrocytes were labeled for 10 min at 37 °C with 2 µM carboxyfluorescein diacetate, succinimidyl ester (CFDA, SE) (Solarbio Life Science, Beijing, China) in Dulbecco’s PBS (D-PBS; Thermo Fisher Scientific) supplemented with 2% FBS (Thermo Fisher Scientific). The same volume of ice-cold D-PBS with 10% FBS was then added to stop the reaction. CFDA, SE is catalyzed into CFSE by intracellular esterase. Then, erythrocytes were washed twice with Mg^2+^/Ca^2+^-free PBS. After centrifugation at 3000 rpm for 5 min, the density of erythrocytes was adjusted and added to the medium with microglia and Tregs at a ratio of 1:10. The percentage of microglia that became CFSE^+^ was analyzed by immunofluorescence.

### Protein preparation and Western blotting

To investigate the effects of SIRPα variants on the signaling pathways after ICH, Western blotting was performed as described previously [15]. Briefly, primary cultured microglia grown in 6-well plates were harvested 24 h after administration with or without erythrocytes and then homogenized in cold RIPA buffer (Cell Signaling Technology) containing 1 mmol/L phenylmethylsulfonyl fluoride (PMSF) and a phosphatase inhibitor cocktail (1:50, Sigma-Aldrich). Simultaneously, the homogenate was centrifuged at 12,000 rpm for 15 min at 4 °C, and the supernatant was collected for protein detection. Twenty-five micrograms of proteins were loaded into each lane and subjected to sodium dodecyl-sulfate polyacrylamide gel electrophoresis (SDS-PAGE) using 4 ~ 15% Ready Gel (Bio-Rad Laboratories Inc.) at 100 V for 120 ~ 180 min. Proteins were transferred to polyvinylidene fluoride (PVDF) membranes (Millipore) at 250 mA for 2 ~ 4 h. The PVDF membranes were incubated overnight with primary antibodies at 4 °C, followed by HRP-labeled secondary antibody (Invitrogen) for 1 h at room temperature. All the primary antibodies used are listed in Table S2. Membranes were scanned using the Typhoon Trio System (GE Healthcare). The optical densities of all protein bands were analyzed by IMAGEQUANT 5.2 software (GE Healthcare).

### Statistical analysis

GraphPad Prism 8.0 software (GraphPad Software Inc., La Jolla, CA, USA) was used to perform statistical analyses. Student’s t test was employed to compare two groups. One-way or two-way analysis of variance (ANOVA) was used for making multiple comparisons, followed by the post hoc test. *P* < 0.05 was considered statistically significant. All data are expressed as the mean ± standard deviation (SD).

## Results

### Construction and identification of SIRPα variants

**Fig. 1:**
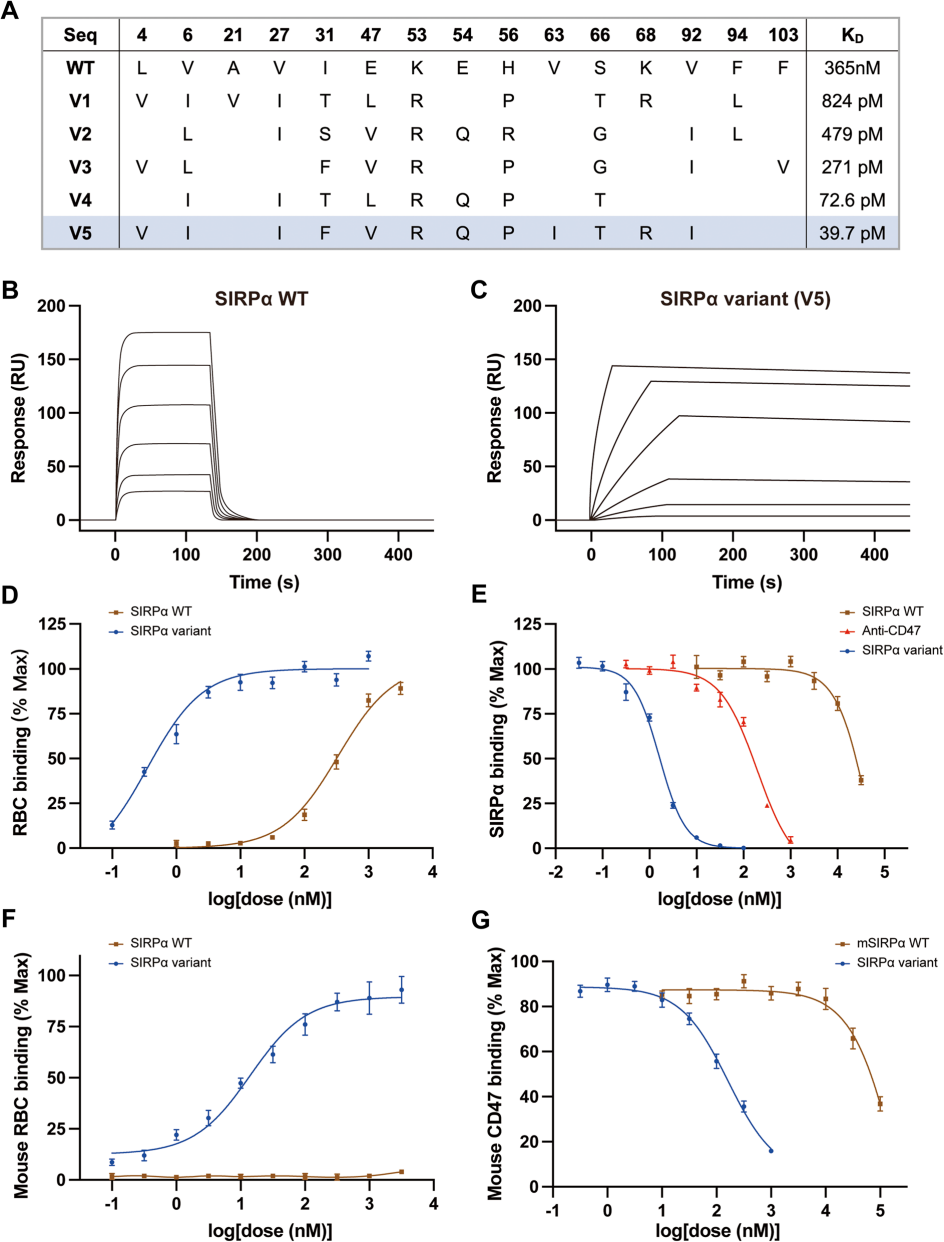
Characterization of high-affinity SIRPα variants. **A** Table of engineered SIRPα variants sequences and affinity measurements. The sequence of wild-type SIRPα (SIRPα WT) and the position of the mutated amino acids are illustrated in the table. The blue shading indicates the final selected type of SIRPα variant. **B** Representative SPR sensorgram of SIRPα WT binding CD47. RU = response units. **C** Representative SPR sensorgram of selected high-affinity SIRPα variant (V5) binding CD47. RU = response units. **D** Titration curves of SIRPα WT (brown) and high-affinity SIRPα variants (SIRPα variant, blue) binding to erythrocytes. **E** Dose–response curves of CD47 antagonism on erythrocytes with SIRPα WT (brown), CD47 antibody clone B6H12 (anti-CD47, red), and V5 SIRPα variant (SIRPα variant, blue). Cells were stained with different concentrations of CD47 blocking agents in competition with Alexa Fluor 594-conjugated wild-type SIRPα tetramer. **F** Fitting curves of SIRPα variant (blue) and SIRPα WT (brown) binding to mouse erythrocytes. Binding assays of biotinylated SIRPα WT and variants were performed with Alexa Fluor 594-conjugated streptavidin. **G** Mouse CD47 blocking assay. SIRPα variant (blue) and wild-type mouse SIRPα (mSIRPα WT, brown) block Alexa Fluor 594-conjugated wild-type mouse SIRPα tetramers binding to mouse CD47 displayed on the surface of yeast. All the data are presented as the mean ± SD

The N-terminal V-set Ig domain of SIRPα or variants was conjugated to Aga2p on the surface of yeast. After constructing libraries of these domains, CD47 IgSF domain-mediated selection was performed to acquire SIRPα variants with high affinity in vitro (Fig. 1A). Selected by SPR, we obtained SIRPα variants (V5) that bound CD47 with dissociation constants (K_D_) as low as 39.7 pM compared to that of wild-type SIRPα (SIRPα WT) for 365 nM (Fig. 1B, C). Moreover, SIRPα variants (V5) presented 40 min dissociation half-lives (t_1/2_) relative to 2.4 s (t_1/2_) for SIRPα WT.

Then, the functional characteristics of the high-affinity SIRPα variants were assessed by binding and blocking analyses in vitro. We found that SIRPα variants with increased CD47 affinity exhibited greater potency in binding and blocking erythrocyte-surface CD47 (Fig. 1D), indicating an enhanced potential of antagonism compared to SIRPα WT. Another test for the anti-CD47 antibody clone B6H12, a well-recognized CD47 antagonist with therapeutic efficacy in vitro and in vivo [22, 38], was also performed. The high-affinity SIRPα variants exhibited more potent antagonism relative to the anti-CD47 antibody in competition with wild-type SIRPα (Fig. 1E), suggesting that the potential of high-affinity variants was superior to that of the anti-CD47 antibody.

Since human exclusively anti-CD47 reagents rarely target mouse CD47 [39], we labeled the high-affinity SIRPα variants with biotin and tested the ability to bind and block mouse CD47. Displayed on the surface of yeast, mouse CD47 was bound to biotinylated high-affinity SIRPα variants, rather than the wild-type human SIRPα, demonstrating that high-affinity SIRPα variants acquired cross-reactivity with mouse CD47 (Fig. 1F). Moreover, the high-affinity SIRPα variant blocked the binding of wild-type mouse SIRPα to mouse CD47, implying that the high-affinity SIRPα variant could serve as an efficacious competitor to wild-type mouse SIRPα (Fig. 1G). Taken together, these results indicated that high-affinity SIRPα variants were capable of binding and blocking mouse CD47. Thus, the V5 type of SIRPα variants was selected and allowed for the evaluation of efficacy and toxicity in subsequent experiments.

### Characterization of SIRPα-v Exos

**Fig. 2:**
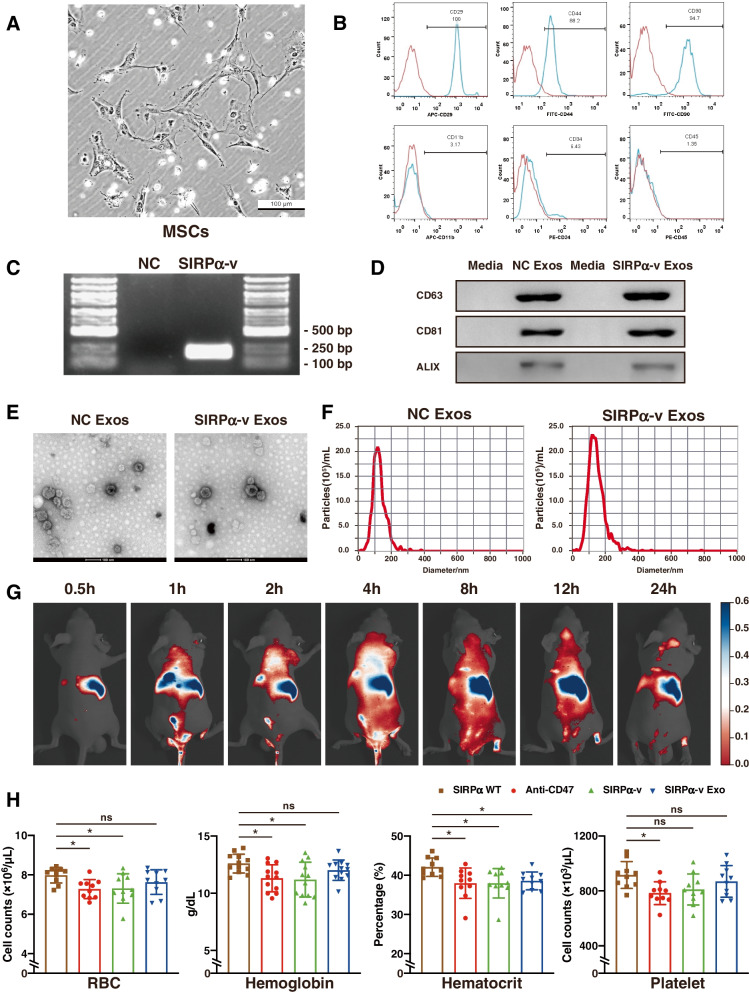
Identification of MSCs and exosomes. **A** Primary cultivation of MSCs. Spindle-shaped or irregular polygonal cells grew in clusters, relatively sparse between the clusters (scale bar = 100 µm). **B** The expression of protein markers on the surface of MSCs. The expression of MSC surface markers. MSCs are CD11b^−^/CD34^−^/CD45^−^/CD29^+^/CD44^+^/CD90^+^ cells. **C** PCR product of SIRPα variant primers on agarose gel electrophoresis. Transfected MSCs stably express SIRPα variants. **D** Western blot assay of CD63, CD81, and Alix. Both empty exosomes (NC Exo) and SIRPα variant-modified exosomes (SIRPα-v Exo) expressed the three biomarkers. **E** Representative TEM images of NC Exos and SIRPα-v Exos. NC Exos and SIRPα-v Exos appeared as double-concave disc-shaped vesicles with different diameters, and low-density bright areas could be observed in the vesicles (scale bar = 100 nm). **F** Fitting curves of NTA analysis. Diameter distribution of NC Exos (diameter: 96.8 ± 5.7 nm) and SIRPα-v Exos (diameter: 100.7 ± 7.3 nm) (*n* = 5). G Representative in vivo fluorescence images of time-dependent biodistribution of DiR-labeled SIRPα-v Exos in mice (*n* = 7). H Analysis of blood cell parameters from SIRPα WT, CD47 antibody (anti-CD47), SIRPα variants (SIRPα-v), or SIRPα-v Exo-treated animals (*n* = 8). All the data are presented as the mean ± SD

First, high-affinity SIRPα variants were engineered. For this purpose, primary cultured MSCs were isolated from the bone marrow of C57BL/6 mice. After cultivation for 7 ~ 10 days, MSCs with a spindle-shaped morphology were achieved (Fig. 2A). To avoid the contamination of hematopoietic stem cells or other cells, flow cytometry was conducted to confirm the purity of MSCs. The results showed that the positive rates of CD29, CD44, and CD90 were 88.2 ~ 100%, while the percentages of CD11b-, CD34-, and CD45-positive cells were all lower than 7% (Fig. 2B). The results suggested that the primary cultured MSCs accounted for approximately 90% of the cells, achieving the purity required for subsequent experiments.

After determining the plasmid sequences of the engineered SIRPα variant and the restriction sites (Fig. S1), we packed and isolated lentivirus from 293 T cells (Fig. S2). Then, the lentiviral sequences of the SIRPα variant were transfected into the primary cultured MSCs (Fig. S3). The results of polymerase chain reaction (PCR) showed that only MSCs infected by engineered lentivirus expressed the sequence of the SIRPα variant (Fig. 2C). The time-dependent expression of SIRPα variant was examined by western blotting (Fig. S4). After 7 days of culture, the supernatant was collected, and exosomes were separated by ultracentrifugation. Western blotting was performed to detect the surface marker proteins of exosomes, and the expression levels of CD63, CD81, and Alix were all positive in both original MSC-exosomes (namely NC Exo) and SIRPα-v Exos (Fig. 2D). TEM revealed that both NC Exos and SIRPα-v Exos were roughly spherical entities with different diameters (Fig. 2E). As measured by nanoparticle tracking analysis (NTA), the mean diameters of these vesicles were 107.8 ± 7.1 nm in the NC Exo group and 117.7 ± 6.3 nm in the SIRPα-v Exo group (Fig. 2F). The NTA results for other batches of exosomes are shown in Fig. S5. These results confirmed that MSC-exosomes could be successfully prepared with the typical morphology and molecular features of exosomes, and both NC Exos and SIRPα-v Exos could be utilized in subsequent studies.

To display the time-dependent biodistribution after SIRPα-v Exo administration we labeled the exosomes with DiR and recorded their biodistribution at different time points by in vivo fluorescence imaging system. As shown in Fig. 2G, SIRPα-v Exos were observed in the liver 30 min after intravenous injection. SIRPα-v Exos were first distributed in the liver and then dispersed to the kidney, lung, and other tissues and organs. Aggregation of SIRPα-v Exos were detected in the brain 8 h after injection. Although SIRPα-v Exos were diminished, part of the fluorescence signal of SIRPα-v Exos remained in the brain 24 h after injection. Moreover, major organs were examined in vitro (Fig. S6A). Additional examination of the BBB permeability of SIRPα-v Exos was performed in mice after ICH. Compared to normal mice, the signal intensity of SIRPα-v Exos in the brain was increased after ICH, exceeding 5% (Fig. S6B). Immunofluorescence of brain tissue demonstrated SIRPα-v Exo penetration of the BBB (Fig. S7). We also examined the release of SIRPα-v from SIRPα-v Exos in vitro and in vivo. The interaction between SIRPα-v and erythrocytes indicated that SIRPα-v Exos not only crossed the BBB but also released SIRPα-v that bound to erythrocytes (Fig. S8). In addition, we observed that some microglia were labeled with PHK26 as a result of the interaction of SIRPα-v Exos and microglia (Fig. S9).

Due to CD47 expression on blood cells, we used SIRPα WT monomer, CD47 antibody (clone B6H12), SIRPα variant monomer (SIRPα-v), and SIRPα-v Exos to test the biosafety in mouse models. After 14-day consecutive intravenous injection, whole blood cells of mice were analyzed. Our data showed that SIRPα-v covered all blood cells and caused anemia as a side effect of the treatment. In contrast, no hematologic toxicity was observed in SIRPα-v Exo-treated mice (Fig. 2H). Anemia was also detected in mice treated with CD47 antibodies, which is consistent with other findings [40]. Analysis of the other components of circulating blood cells is displayed in Table S1. To further clarify the toxicity of SIRPα-v Exos in other aggregated organs, we performed more in-depth assays. According to serological results, the liver and kidneys showed mild injury after continuous administration of SIRPα-v Exos, while no injury was detected in the heart (Fig. S10A, B, C, D and E). No corresponding evidence of injury was found histologically in either the liver or kidneys (Fig. S11). Changes in serum biomarkers disappeared after SIRPα-v Exo cessation, suggesting that these injuries were most likely functional rather than organic. As a drug for intravenous application, vascular toxicity should not be ignored. Related markers of vascular endothelial injury such as soluble thrombomodulin (sTM) and von Willebrand factor (vWF) in serum have been examined as well. The results revealed a slight upward expression trend of sTM and vWF without statistical significance (Fig. S12).

### SIRPα-v Exos accelerate the clearance of hematoma and preserve neurological dysfunction of ICH

**Fig. 3:**
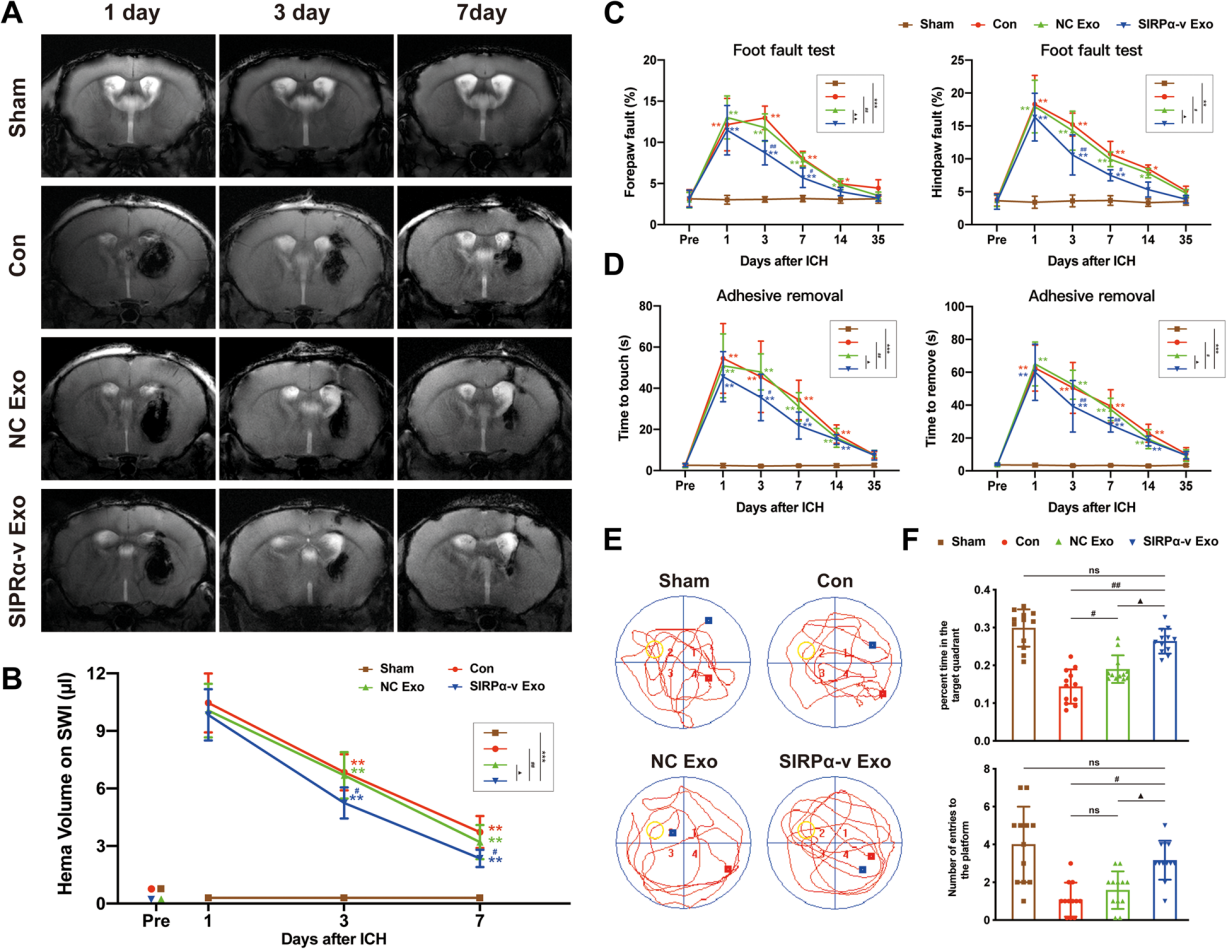
SIRPα-v Exos accelerate the clearance of hematoma and improve long-term prognosis after ICH. **A** Coronal SWI images of radiological hematoma changes in mice after ICH, assessed by 11.7 T Ultrahigh field magnetic resonance on the 1st, 3rd, and 7th days post-ICH. **B** Quantification analysis of the hematoma volume on the 1st, 3rd, and 7th days post-ICH. Compared with the Con and NC Exo groups, the clearance of hematoma was markedly accelerated in the SIRPα-v Exo group (*n* = 12/group). **C**, **D** Sensorimotor functions assessed before and 1, 3, 7, 14, and 35 days after ICH or sham surgery. **C** The foot-fault test. The frequency of the forepaw or hindpaw was quantified (*n* = 12/group). **D** The adhesive removal test. The latency of biting or licking to remove the sticker was measured (*n* = 10/group). **E** Representative images of swim paths of mice in each group evaluated by the Morris water maze test on the 35th day post-ICH. The yellow circles represent the location where the platform was previously located. **F** The time spent in the quadrant and frequency of visits to the quadrant of the previously located platform (*n* = 12/group). * *P* < 0.05, ** *P* < 0.01, *** *P* < 0.001 *vs.* Sham group; # *P* < 0.05, ## *P* < 0.01 *vs.* Con group; ▲ *P* < 0.05, *vs*. NC Exo group; ns, no significance. All the data are presented as the mean ± SD

To investigate whether SIRPα-v Exos accelerate the elimination of hematoma after ICH, we intravenously injected exosomes into mice suffering from ICH through the tail vein for 14 consecutive days at a concentration of 6 mg/kg. On the 1st, 3rd, and 7th days post-ICH, mice were scanned with susceptibility weighted imaging (SWI) sequence by an 11.7 T ultrahigh field MRI system (Fig. 3A). Additionally, corresponding images of hematoxylin and eosin stain (H.E.) staining are displayed in Fig. S13. These results revealed that the volume of intracerebral hematoma in mice that received SIRPα-v Exo treatment (SIRPα-v Exo group) was smaller than that in mice in the control group (Con group) and original MSC-exosomes group (NC Exo group) on the 3rd and 7th days post-CH (Fig. 3B), which indicated that SIRPα-v Exo promoted the clearance of hematoma.

To determine the effects of SIRPα-v Exo treatment on motor and sensorimotor functions, foot-fault and adhesive removal tests were applied on the 1st, 3rd, 7th, 14th, and 35th days post-ICH. The results of the foot-fault test showed that the error ratio of both the forelimbs and hindlimbs in paw placement on grid-walking was markedly lower in the SIRPα-v Exo group than in the Con and NC Exo groups (Fig. 3C). Specifically, improvement of forelimbs seemed to be more noticeable. Moreover, the results of the adhesive removal test indicated that the time to touch the paw and remove the sticker was shortened in the SIRPα-v Exo group (Fig. 3D). In addition, improvements confirmed by both foot-fault and adhesive removal tests were observed on the 3rd day post-ICH.

To further explore whether SIRPα-v Exo treatment improves long-term neurological complications in mice after ICH, we performed the Morris water maze test 35 days after ICH (Fig. 3E). Compared with the Con group, when the platform was removed, the time spent in the target quadrant increased in the NC Exo and SIRPα-v Exo groups, while the number of entries to the platform only increased in the SIRPα-v Exo group (Fig. 3F). Other typical analyses are displayed in Fig. S14. The results of the open-field test (OFT), tail suspension test (TST) and forced swim test (FST) showed that SIRPα-v Exos ameliorated depressive symptoms such as nervousness, anxiety and fear exhibited in mice after ICH (Fig. S15). NC Exos also performed an ameliorating effect. All the results of neurobehavioral test results suggested that the NC Exo- and SIRPα-v Exo-treated groups exhibited long-term improvements in cognitive function and depressive-like behaviors, with SIRPα-v Exo treatment being more effective. The abovementioned results demonstrated that SIRPα-v Exo treatment improved short-term and long-term neurological complications in mice after ICH.

### SIRPα-v Exos alleviate WMI after ICH

**Fig. 4:**
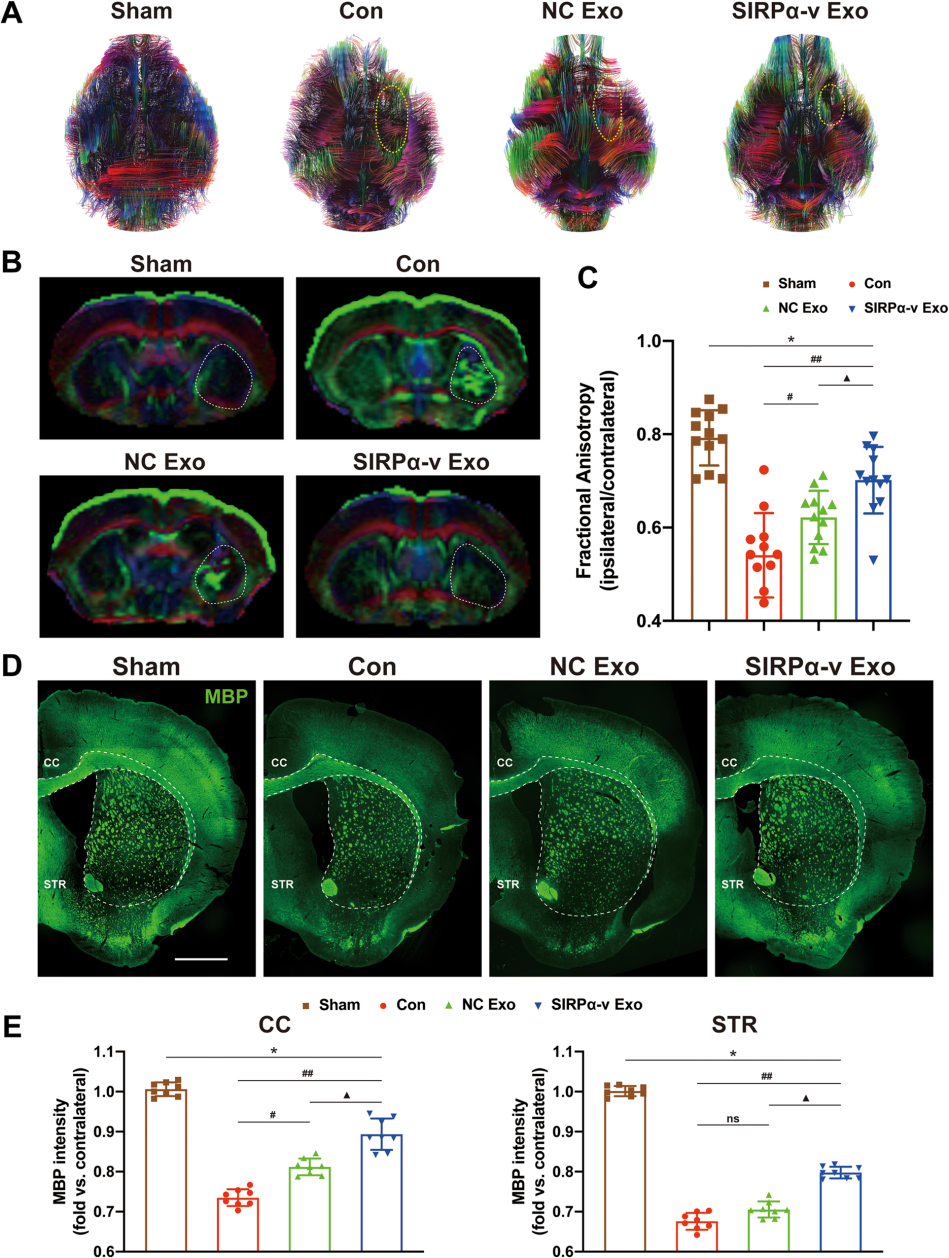
SIRPα-v Exos alleviate WMI after ICH. **A** Representative images of 3D reconstruction of DTI on the 35th day post-ICH. The areas in the yellow dotted lines show the defects of white matter tracts. **B** Representative coronal images of the DEC map on the 35th day post-ICH. **C** Quantification of FA values on the ipsilateral STR on the 35th day post-ICH. Data are expressed as the ratio of FA values in the ipsilateral (lesioned) hemispheres to the FA values in the non-lesioned contralateral hemispheres (*n* = 12/group). **D** Representative images of MBP (green) immunofluorescence in the area associated with DTI (scale bar = 1 mm). **E** Quantification of the fluorescence intensity (CC and STR) in the area around the hematoma. Data are calculated as fold-change compared to the corresponding contralateral areas (*n* = 6/group). * *P* < 0.05, ** *P* < 0.01 *vs.* Sham group; # *P* < 0.05, ## *P* < 0.01 *vs.* Con group; ▲ *P* < 0.05, *vs*. NC Exo group; ns, no significance. All the data are presented as the mean ± SD. CC, corpus callosum, STR, striatum

We next carried out 11.7 T ultrahigh field MR to explore the effects of SIRPα-v Exos on WMI after ICH. As a noninvasive method, diffusion tensor imaging (DTI) is used to effectively trace the nerve fiber bundle and quantitatively evaluate nerve injury [41]. According to the results of three-dimensional (3D) reconstruction of DTI, compared with mice in the Con and NC Exo groups, WMI was relieved in mice in the SIRP-v Exo group on the 35th day post-ICH (Fig. 4A). Further analysis of fractional anisotropy (FA) revealed that NC Exo and SIRPα-v Exo treatments enhanced the structural integrity of white matter, while SIRPα-v Exo treatment performed better than NC Exo treatment (Fig. 4B, C).

As a protein in the myelination process of CNS, myelin basic protein (MBP) maintains the correct structure of myelin and interacts with lipids in the myelin membrane [42]. Therefore, we analyzed MBP by immunofluorescence (Fig. 4D). The MBP fluorescence intensity of both the corpus callosum (CC) and striatum (STR) in mice in the SIRPα-v Exo group was noticeably higher than that in the Con and NC Exo groups after ICH (Fig. 4E). Compared with the DTI findings, the immunofluorescent of MBP showed a trend consistent with the FA values, indicating that the integrity of white matter was preserved by SIRPα-v Exo treatment.

**Fig. 5:**
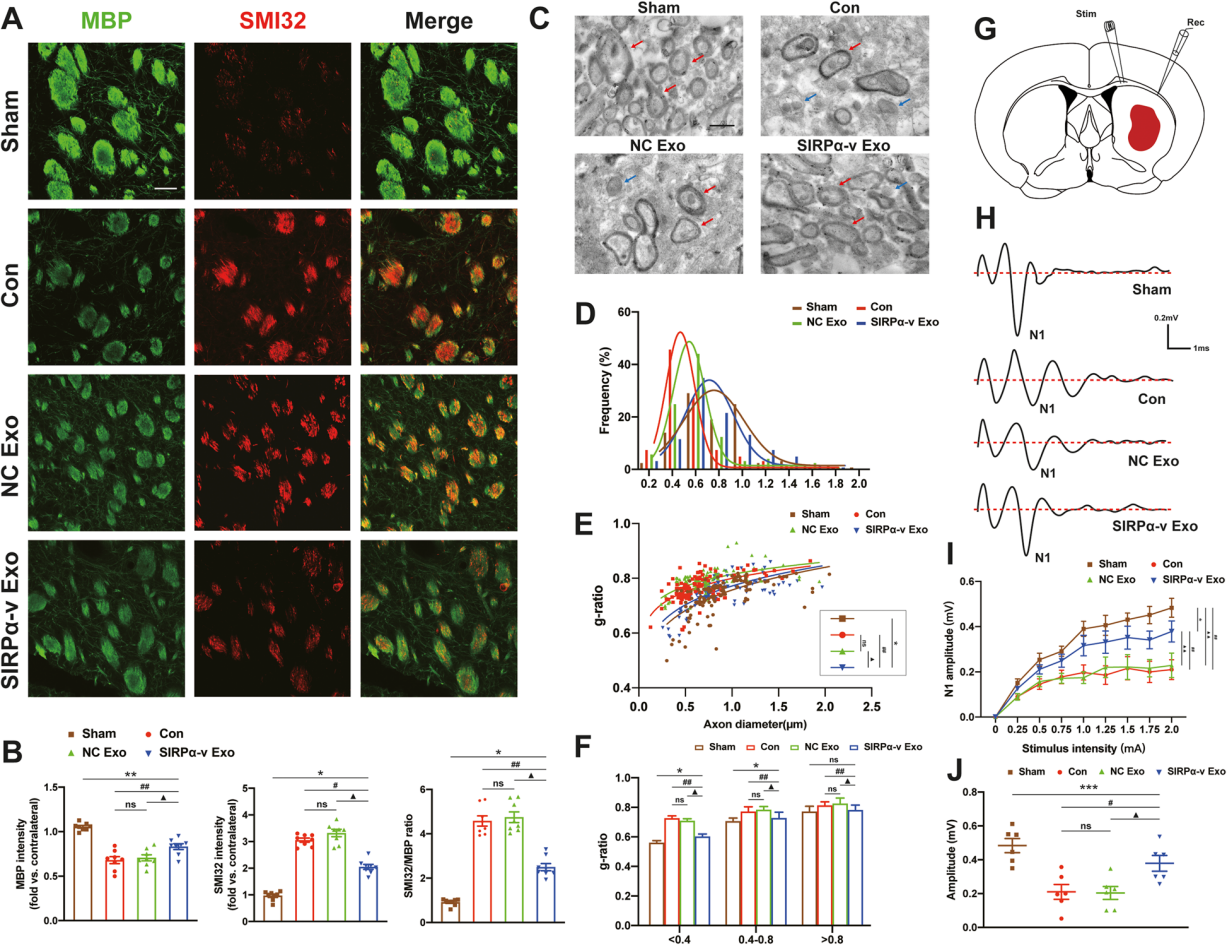
SIRPα-v Exos ameliorate demyelination after ICH. **A** Representative immunofluorescence images of MBP and SMI32 in the ipsilateral STR on the 35th day post-ICH (scale bar = 100 μm). **B** Quantification of the fluorescence intensities of MBP and SMI32 and the ratio of SMI32 to MBP intensity in the ipsilateral STR (*n* = 8/group). Data are normalized to the intensities of contralateral hemispheres. **C** Representative TEM images of myelin integrity in the ipsilateral STR on the 35th day post-ICH or after sham surgery (scale bar = 1000 nm). Red arrows indicate medullated fibers, and blued arrows indicate damaged medullated fibers. **D** Frequency histograms of all the quantified normally myelinated axons showing the distribution of axon diameter. **E** Scatter plots of the g-ratio versus axon diameter in the sham-operated group or all ICH groups on the 35th day. **F** The g-ratio of myelinated axons with respect to the axon diameter at 0.4-μm intervals in the sham-operated group or all ICH groups on the 35th day post-ICH (*n* = 5/group). **G** Schematic illustration of the position of the stimulating (Stim) and recording (Rec) electrodes for CAP measurements in the CC/EC. **H** Representative traces of the evoked CAPs in the CC (stimulus, 2 mA; 0.48 mm lateral to the stimulating electrode) on the 35th day post-ICH. **I** Quantification of the amplitude of the evoked CAPs of myelinated N1 fibers in response to the increase in stimulus strength (0.0 ~ 0.20 mA). **J** N1 amplitude in response to a 0.2 mA stimulus on the 35th day post-ICH (*n* = 6/group). * *P* < 0.05, ** *P* < 0.01, *** *P* < 0.001 *vs.* Sham group; # *P* < 0.05, ## *P* < 0.01 *vs.* Con group; ▲ *P* < 0.05, ▲▲ *P* < 0.01 *vs*. NC Exo group; ns, no significance. All the data are presented as the mean ± SD

Dephosphorylated neurofilament protein (SMI32) recognizes nonphosphorylated neurofilaments and can be found in damaged axons [42]. To determine whether SIRPα-v Exo treatment could improve axonal injury after ICH, double immunofluorescence of MBP and SMI32 was performed on the 35th day post-ICH (Fig. 5A). The overall immunofluorescence intensity of MBP decreased in mice suffering from ICH, indicating a large loss of myelin after ICH. In addition, the overall immunofluorescence intensity of SMI32 increased and led to a notable elevation in the SMI32/MBP ratio after ICH. Compared with mice in the Con and NC Exo groups, the SMI32/MBP ratio decreased in mice in the SIRPα-v Exo group (Fig. 5B). These results demonstrated that SIRPα-v Exo treatment protected against axonal injury after ICH.

The thickness of myelin sheath in the STR was measured by TEM (Fig. 5C). The frequency histogram showed that the axon diameters in the Con and NC Exo groups were reduced compared with those in the SIRPα-v Exo group on the 35th day post-ICH (Fig. 5D). Mice that did not receive SIRPα-v Exo treatment presented more severe axonal degeneration. The ratio of the inner-to-outer diameter of a myelinated axon (g-ratio) reflects axonal function and integrity. The scatter plot of axon diameter against the g-ratio illustrated that the thickness of myelin sheath was higher in the SIRPα-v Exo group on the 35th day post-ICH than in the other groups (Fig. 5E). The g-ratio of small axonal fibers (diameter < 400 nm), medium axonal fibers (diameter = 400 ~ 800 nm), and large axonal fibers (diameter > 800 nm) decreased in the SIRPα-v Exo group compared with the Con and NC Exo groups. Moreover, the g-ratio in large axonal fibers (diameter > 800 nm) showed a greater improvement than that in other axonal fibers (Fig. 5F). These results suggested that SIRPα-v Exos protected axonal myelin after ICH.

As SIRPα-v Exos were able to protect the structural integrity of white matter after ICH, we evaluated the function of white matter by measuring the transmission of CAPs on the 35th day post-ICH (Fig. 5G). The evoked CAPs showed an early peak, which represented fast transmission of myelinated axons [42]. The amplitude of the N1 segment was reduced after ICH, which indicated that conduction through myelinated axons was impaired (Fig. 5H). A lower reduction in the amplitude of the N1 segment was detected in mice in the SIRPα-v Exo group after ICH than in mice in the other groups (Fig. 5I, J). Thus, SIRPα-v Exos preserved the myelin sheaths of axons.

### SIRPα-v Exos modulate the polarization of microglia/macrophages

**Fig. 6:**
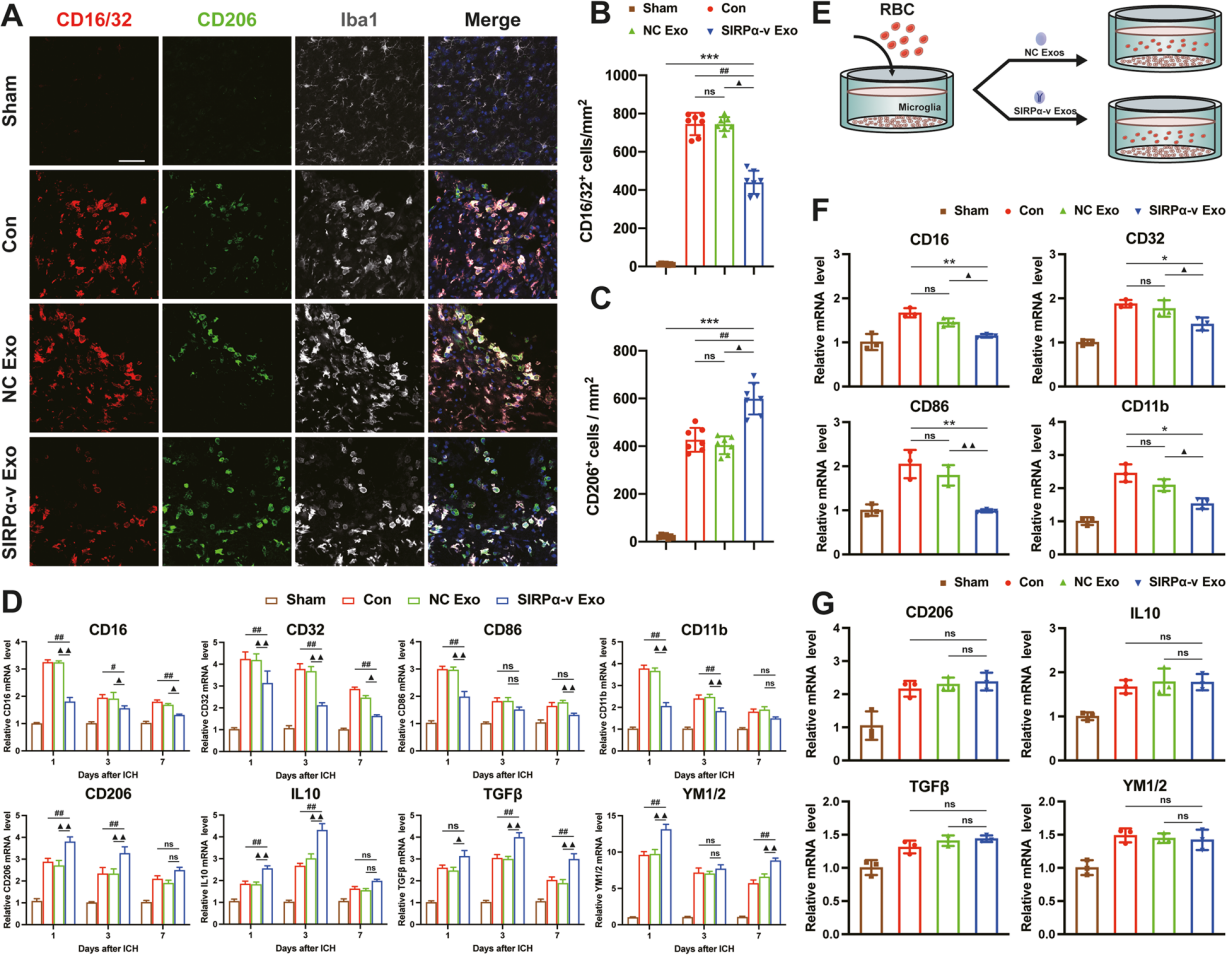
SIRPα-v Exos modulated the polarization of microglia/macrophages after ICH. **A** Representative immunofluorescence images of CD16/32 (red), CD206 (green), and Iba1 (silver) in the ipsilateral STR around the hematoma on the 3rd day post-ICH (scale bar = 100 μm). **B** Quantification analysis of CD16/32^+^ and Iba1^+^ cells in the ipsilateral STR around the hematoma on the 3rd day post-ICH. **C** Quantification analysis of CD206^+^ and Iba1^+^ cells in the ipsilateral STR around the hematoma on the 3rd day post-ICH. **D** The mRNA levels of CD16, CD32, CD86, and CD11b (biomarkers for M1 microglia) and CD206, IL-10, TGFβ, and YM1/2 (biomarkers for M2 microglia) detected by RT-qPCR on the 1st, 3rd, 7th days post-ICH (*n* = 6/group). **E** Schematic illustration of the experimental design (separate application of SIRPα-v Exos) in vitro. **F** The mRNA levels of CD11b, CD16, CD32, and CD86 detected by RT-qPCR after separate application of SIRPα-v Exos (*n* = 3/group). **G** The mRNA levels of CD206, TGFβ, IL-10, and YM1/2 detected by RT-qPCR after separate application of SIRPα-v Exos (*n* = 3/group). * *P* < 0.05, ** *P* < 0.01, *** *P* < 0.001 *vs.* Sham group; # *P* < 0.05, ## *P* < 0.01 *vs.* Con group; ▲ *P* < 0.05, ▲▲ *P* < 0.01 *vs*. NC Exo group; ns, no significance. All the data are presented as the mean ± SD

We analyzed the polarization of microglia/macrophages in the STR after ICH to determine whether SIRPα-v Exos could regulate the polarization of microglia/macrophages by affecting the CD47-SIRPα interaction. Brain slices were subjected to triple immunofluorescence with M1-phenotype markers (CD16/32), M2-phenotype marker (CD206), and Iba1 (a macrophage/microglia-specific calcium-binding protein) on the 3rd day post-ICH (Fig. 6A).

Compared with the sham-operated group, the number of cells expressing CD16/CD32 and CD206 in the Con, NC Exo, and SIRPα-v Exo groups was remarkably elevated. However, an increase in the number of CD206-positive cells and a decrease in the number of CD16/32-positive cells were detected in mice in the SIRPα-v Exo group compared with those in the Con and NC Exo groups (Fig. 6B, C). The results of immunofluorescence with Iba1 showed that SIRPα-v Exos regulated the polarization of microglia/macrophages and promoted their polarization to the M2-phenotype. We also used RT-qPCR to examine the alterations of microglia/macrophages in STR. The expression levels of M1-phenotype markers (CD16, CD32, CD86, and CD11b) and M2-phenotype markers (CD206, interleukin-10 (IL-10), transforming growth factor-β (TGF-β), and YM1/2) were remarkably upregulated, indicating that microglia/macrophages were activated after ICH (Fig. 6D). In line with immunofluorescence, the results of RT-qPCR showed that the expression levels of CD16, CD32, CD86, and CD11b were downregulated in the SIRPα-v Exo group compared with the Con and NC Exo groups, which reflected a reduction in M1 microglia/macrophage polarization. Simultaneously, the expression levels of CD206, IL-10, TGF-β, and YM1/2, reflecting the M2 polarization of microglia/macrophages, were upregulated after SIRPα-v Exo treatment (Fig. 6D). SIRPα-v Exos promoted the polarization of microglia/macrophages toward the M2-phenotype. These results demonstrated that SIRPα-v Exo regulated the expression levels of related markers at both the transcriptional and translational levels.

To further evaluate the effects of SIRPα-v Exos on the polarization of microglia/macrophages in vitro, we also added NC Exos or SIRPα-v Exos to the medium to assess changes in the polarization of primary microglia cocultured with erythrocytes (Fig. 6E, Fig. S16). The results of RT-qPCR revealed that primary microglia exhibited lower expression levels of CD16, CD32, CD86, and CD11b in the SIRPα-v Exo group than in the Con and NC Exo groups (Fig. 6F). In agreement with the results of in vivo experiments, these data indicated that M1 microglial polarization was reduced by SIRPα-v Exos. However, the expression levels of CD206, IL-10, TGF-β, and YM1/2 did not change significantly (Fig. 6G), revealing that SIRPα-v Exos did not directly regulate the M2 polarization of microglia.

### Tregs are required for SIRPα-v Exos to promote M2 polarization and phagocytosis of microglia

**Fig. 7:**
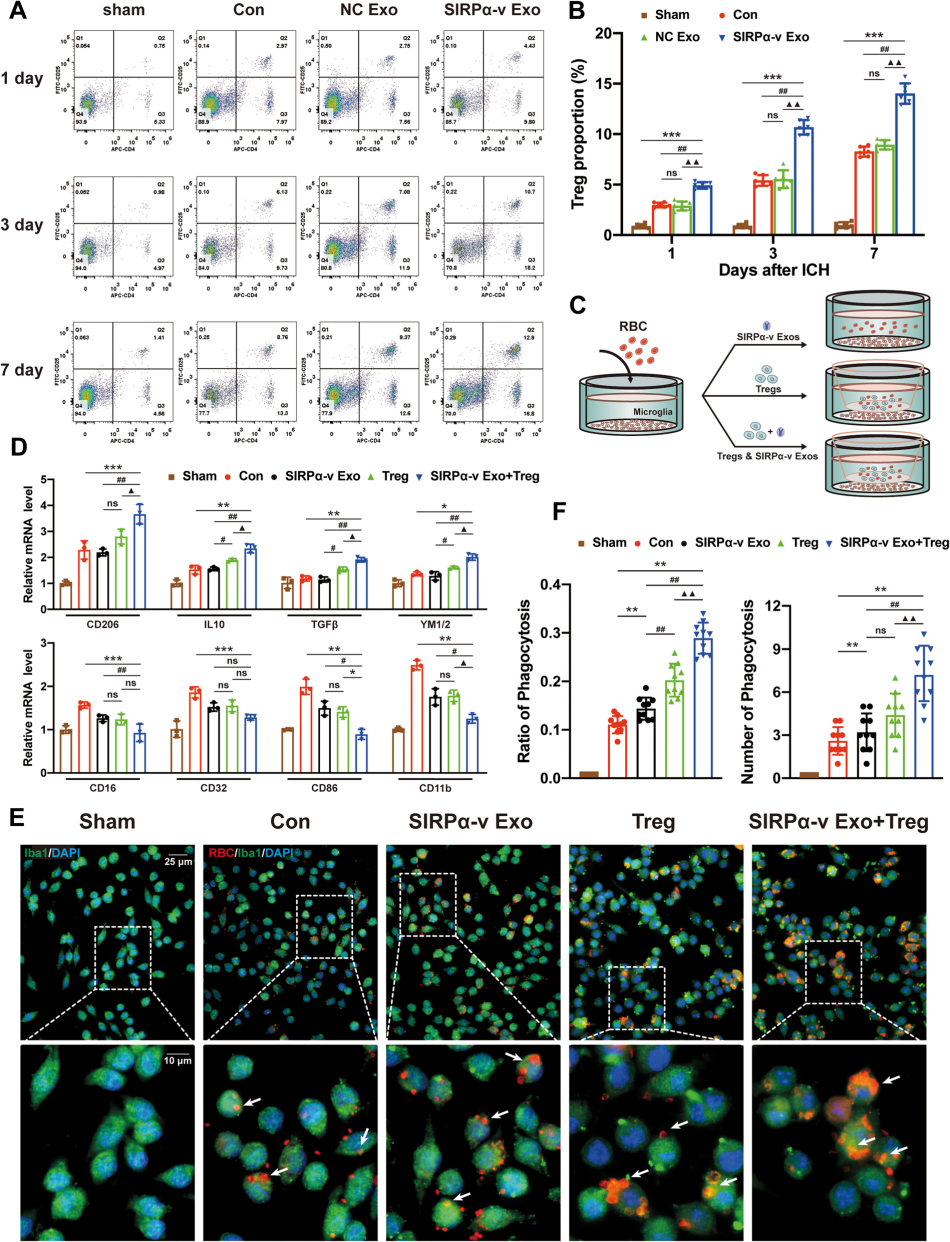
Tregs were required for promoting the M2-phenotype and phagocytosis by SIRPα-v Exos. **A** Representative flow cytometry images of CD4^+^ CD25^+^ cells in the ipsilateral STR around the hematoma on the 1st, 3rd, and 7th days post-ICH (*n* = 6/group). **B** Changes in the proportion of CD4^+^ CD25^+^ cells in the ipsilateral STR around the hematoma on the 1st, 3rd, and 7th days post-ICH (*n* = 6/group). * *P* < 0.05, ** *P* < 0.01, *** *P* < 0.001 *vs.* Sham group; # *P* < 0.05, ## *P* < 0.01 *vs.* Con group; ▲ *P* < 0.05, ▲▲ *P* < 0.01 *vs*. NC Exo group; ns, no significance. **C** Schematic illustration of the experimental design (combined application of Tregs and SIRPα-v Exos) in vitro. **D** The mRNA levels of CD206, TGFβ, IL-10, YM1/2, CD11b, CD16, CD32, and CD86 assessed by RT-qPCR after administration of Tregs or a combination of Tregs and SIRPα-v Exos (*n* = 3/group). **E** Representative immunofluorescence images of erythrocytes (red), Iba1 (green), and DAPI (blue) in primary microglia treated with or without Tregs and SIRPα-v Exos (scale bar_1_ = 25 μm, scale bar_2_ = 10 μm). The white arrows indicate microglia that phagocytosed erythrocytes. **F** Quantification analysis of the ratio of microglia phagocytosing erythrocytes in each field of view and erythrocytes phagocytosed per microglia 24 h after coculture with erythrocytes (*n* = 10/group). * *P* < 0.05, ** *P* < 0.01, *** *P* < 0.001 *vs.* Con group; # *P* < 0.05, ## *P* < 0.01 *vs.* SIRPα-v Exo group; ▲ *P* < 0.05, ▲▲ *P* < 0.01 *vs*. Treg group; ns, no significance. All the data are presented as the mean ± SD

In previous experiments, we explored the effects of SIRPα-v Exos on the polarization of primary microglia in vitro. Contrary to the results detected in vivo, the results of RT-qPCR showed that SIRPα-v Exos failed to exert regulatory effects on M2 microglia independently, indicating that other pathways or immunocytes could be involved in the regulation of M2 microglia. Exploiting the knowledge of the immune response between microglia/macrophages and T cells in CNS diseases, regulatory T cells (Tregs) were identified [43–45]. We counted Tregs in the striatal tissue surrounding the hematoma. For this purpose, flow cytometry was undertaken to detect Tregs in STR on the 1st, 3rd, and 7th days post-ICH (Fig. 7A). The number of CD4^+^ CD25^+^ cells in peri-hematoma tissue gradually increased from the 1st day, and this trend was maintained until 7 days after ICH. SIRPα-v Exos incurred more significant increase in the number of CD4^+^ CD25^+^ cells than that in the Con and NC Exo groups (Fig. 7B). The abovementioned findings demonstrated that SIRPα-v Exo treatment recruited Tregs in the striatal tissue surrounding the hematoma in the acute phase of ICH.

Considering the recruitment of Tregs in the peri-hemorrhagic region of the STR, the combined application of Tregs and SIRPα-v Exos was carried out to determine whether SIRPα-v Exos regulated microglia in association with Tregs in vitro (Fig. 7C). Mouse spleens were dissected to collect CD4^+^ CD25^+^ cells by the MACS method, which simulated Tregs in vitro (Fig. S17). After co-treatment with Tregs and SIRPα-v Exos, the RT-qPCR results showed upregulated expression levels of CD206, IL-10, TGF-β, and YM1/2 (Fig. 7D). In addition, the expression levels of CD16, CD32, CD86, and CD11b were also downregulated (Fig. 7D). These findings revealed that Tregs are one of the indispensable factors for the regulation of M2 microglial polarization.

To obtain quantitative measurements of phagocytosis, primary microglia and CFSE^+^ erythrocytes were cocultured with SIRPα-v Exos, and the percentage of microglia that became CFSE^+^ was analyzed by immunofluorescence. We compared Tregs, SIRPα-v Exos, and their combination (namely SIRPα-v Exo + Treg) in the administration of microglia. After coculturing for 24 h, we observed that all microglia phagocytosed erythrocytes. The ratio of microglia that phagocytosed erythrocytes increased after the administration of Tregs, SIRPα-v Exos, and a combination of Tregs and SIRPα-v Exos (Fig. 7E). The combined application of Tregs and SIRPα-v Exos increased the phagocytosis of erythrocytes by microglia more significantly than the separate application of SIRPα-v Exos or Tregs (Fig. 7F). Interestingly, the size of particles phagocytosed by untreated or SIRPα-v Exo-treated microglia was smaller than those phagocytosed by Treg- or SIRPα-v Exo + Treg-treated microglia. These smaller particles were probably fragments of lysed erythrocytes. These results suggested that Tregs or a combination of Tregs and SIRPα-v Exos might increase microglial phagocytosis of erythrocytes before their lysis. Moreover, it was noted that separate application of Tregs did not increase the number of erythrocytes phagocytosed by single microglia. In contrast, the combined application of Tregs and SIRPα-v Exos not only increased the ratio of microglia phagocytosed erythrocytes but also increased the number of erythrocytes phagocytosed by single microglia (Fig. 7F), indicating that SIRPα-v Exos enhanced the removal of hematoma associated with Tregs after ICH.

### SIRPα-v Exos regulate microglial polarization via the p38 MAPK-STAT1 and PI3K-Akt-mTOR signaling pathways

**Fig. 8:**
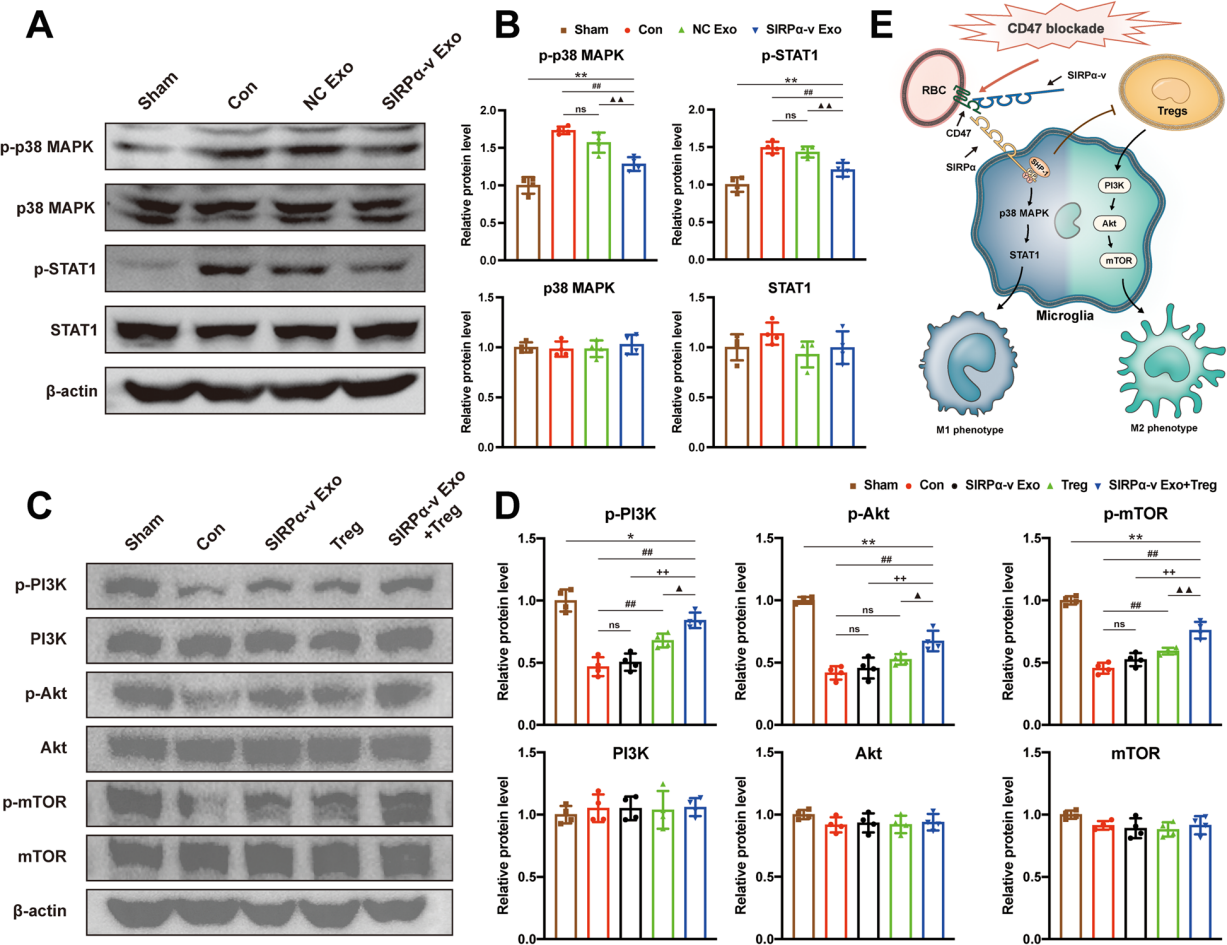
SIRPα-v Exos regulate microglial polarization via the p38-STAT1 and PI3K-Akt-mTOR signaling pathways. **A** Western blot images showing the protein levels of phospho-/nonphospho- p38 MAPK and STAT1 in primary microglia. **B** Quantification analysis of the expression of phospho/nonphospho-p38 MAPK and STAT1 by Western blotting (*n* = 4/group). * *P* < 0.05, ** *P* < 0.01, *vs.* Sham group; # *P* < 0.05, ## *P* < 0.01 *vs.* Con group; ▲ *P* < 0.05, ▲▲ *P* < 0.01 *vs*. NC Exo group; ns, no significance. **C** Representative Western blot images showing the protein levels of phospho/nonphospho-PI3K, Akt, and mTOR in primary microglia. **D** Quantification analysis of the phosphorylation levels of PI3K, Akt, and mTOR by Western blot (*n* = 4/group). **E** Schematic illustration of the proposed mechanisms underlying the regulation of microglia by SIRPα variants. * *P* < 0.05, ** *P* < 0.01, *vs.* Sham group; # *P* < 0.05, ## *P* < 0.01 *vs.* Con group; + *P* < 0.05, +  + *P* < 0.01, *vs.* SIRPα-v Exo group; ▲ *P* < 0.05, ▲▲ *P* < 0.01 *vs*. Treg group; ns, no significance. All the data are presented as the mean ± SD

To delineate the regulatory mechanism of SIRPα-v Exos on primary microglia, we next designed in vitro experiments to investigate the corresponding signaling pathways in primary microglia. As one of the key mechanisms for M1 microglial polarization [46, 47], p38 mitogen-activated protein kinase (MAPK) and signal transducer and activator of transcription 1 (STAT1) signaling pathways were detected by Western blot. Compared with the Con and NC Exo groups, the levels of phospho-p38 MAPK and phospho-STAT1 were downregulated in the SIRPα-v Exo group (Fig. 8A, B). These results indicated that SIRPα-v Exos regulated the polarization of microglia by downregulating the phosphorylation of p38 MAPK, inhibiting the activation of the p38 MAPK-STAT1 signaling pathway, and reducing the polarization of M1 microglia (Fig. 8E).

After the introduction of Tregs, SIRPα-v Exos showed an observable regulatory effect on M2 microglia. Western blotting was also undertaken to investigate the underlying mechanisms. The results showed that the phosphorylation level of phosphatidylinositol 3-kinase (PI3K) in the Treg and SIRPα-v Exo + Treg groups was markedly elevated compared with that in the Con and SIRPα-v Exo groups (Fig. 8C, D). Correspondingly, its downstream proteins Akt and mammalian target of rapamycin (mTOR) exhibited the same trend. The phosphorylation levels of Akt and mTOR were upregulated in the SIRPα-v Exo + Treg group (Fig. 8C, D). However, the phosphorylation levels did not vary in the SIRPα-v Exo group. These results suggested that SIRPα-v Exos regulated M2 polarization of microglia/macrophages associated with Tregs via the PI3K-Akt-mTOR signaling pathway (Fig. 8E).

## Discussion

In the present study, we engineered high-affinity SIRPα variants and applied exosomes for delivery to help reach the lesion more precisely. We found that, first, SIRPα-v Exos accelerated the clearance of hematoma after ICH in mice. To some extent, both unmodified and modified exosomes alleviated WMI and ameliorated neurological dysfunction. Second, SIRPα-v Exos regulated the polarization and biological functions of microglia/macrophages. Although similar to NC Exos, SIRPα-v Exos were superior in the modulation of neuroinflammation and improvement of neurological deficits. The in vitro experiments demonstrated that SIRPα-v Exos enhanced the phagocytosis efficiency of microglia against erythrocytes in synergy with Tregs. Furthermore, our data suggested that SIRPα-v Exos coregulated microglia via both the p38-STAT1 and PI3K-Akt-mTOR signaling pathways.

As one of the subtypes of stroke, ICH is a leading cause of death and disability. Directly destroyed by hematoma, the primary injury of brain tissue is an important factor in causing neurological symptoms. The squeezing effect of the hematoma incurs WMI. Lysis of erythrocytes is recognized as a key factor contributing to secondary brain injury, with more profound impacts than primary injury following ICH [48]. The lysate of erythrocytes leads to a series of complex pathophysiological processes, such as cytotoxic effects and neuroinflammation, exacerbating secondary injury [49]. Early clearance of the hematoma reduces the neuroinflammatory response triggered by erythrocyte lysis products and prevents conversion of WMI from compression or displacement to necrosis, providing an opportunity to mitigate secondary injury.

Under physiological conditions, CD47 is highly expressed by erythrocytes to avoid detection by macrophages through CD47-SIRPα signaling [50]. Mostly, microglia or macrophages perform the clearance of erythrocytes after ICH [36]. Due to these characteristics, microglia/macrophages have difficulty recognizing erythrocytes in a timely manner after ICH. This impairs the clearance of erythrocytes by microglia/macrophages. Blocking inhibitory signaling with antibodies that prevent CD47 from binding to SIRPα boosts macrophage phagocytic capacity [40, 51]. However, antibodies have limited tissue distribution and exert off-target effects [52, 53]. Despite significant efforts by numerous groups, the lack of biosafety-related experiments in previously reported studies has hindered their translation to clinical applications. Accordingly, the goal of our study was to effectively block the interaction between CD47 and SIRPα to boost microglia/macrophages. In our study, we aimed to apply SIRPα as a competitive antagonist of CD47. Unfortunately, the weak affinity of wild-type SIRPα precludes its use as a potential therapy. Therefore, we evolved the affinity of SIRPα by mutating the amino acid sequence to obtain a potent CD47 antagonist. Through repeated in vitro sifting, the final selected high-affinity SIRPα variants bound CD47 with approximately 12,000-fold higher affinity relative to wild-type SIRPα and even exhibited higher binding capacity than the CD47 antibody clone B6H12. Meanwhile, the longer half-life of high-affinity SIRPα variants also offers the possibility of application as a medication in vivo.

Another challenge in medication development is how to deliver pharmaceuticals to specific lesions. SIRPα variant, as a protein, may be limited in vivo, especially for CNS diseases, due to the presence of the BBB. Exosomes, as endogenous nanovesicles, have advantages in medication delivery [27], facilitating SIRPα variants crossing the BBB to intracerebral lesions. Additionally, the membrane integrity of exosomes was expected to mitigate the biotoxicity of SIRPα variants in the peripheral circulation. Genetically editing MSCs, we obtained modified exosomal SIRPα variants. SIRPα-v Exo aggregation was maintained in the brain 8 h after intravenous injection until the next dose. Compared with the lung and heart, SIRPα-v Exos presented a longer retention in the brain, which may be due to the slower circulation of SIRPα-v Exos in the cerebrospinal fluid (CSF) than in peripheral circulation. To test the therapeutic efficacy of the SIRPα-v Exos in a mouse model, the route and schedule of administration, for example, single dose *vs.* repeated doses, were carefully considered. Intravenous infusion was finally selected, which was regarded to be minimally invasive and simple to use. Drug penetrability into the BBB is correlated with brain lesions. As a representative antibiotic for CNS infections, vancomycin is more likely to penetrate the BBB in patients suffering from CNS infection than in those who do not. Moreover, drugs targeting the CNS can be therapeutic even at relatively low concentrations. For instance, vancomycin takes effect at only 3% CSF/serum ratios in patients with severe CNS infections. Similar to many drugs for the CNS, in healthy mice, less than 5% of SIRPα-v Exos were distributed in the brain, whereas the penetration of SIRPα-v Exos into the BBB increased in mice after ICH. Our results confirm that more than 5% of SIRPα-v Exo distribution in the brain improves the prognosis of ICH in mice. SIRPα variants encapsulated by exosomes were less likely to cause anemia and exhibited milder hematotoxicity than naked SIRPα variants and CD47 antibodies. By a series of in vitro and in vivo assays, SIRPα-v Exos exhibited no more severe toxicity than mature anti-CD47 monoclonal antibodies in major organs. By penetrating the BBB more efficiently, SIRPα-v Exos showed excellent potential for short- and long-term safety, establishing a substantial profile of SIRPα-v Exos as preclinical applications. Compared with the Con group, SIRPα-v Exos accelerated hematoma clearance and ameliorated motor and cognitive decline and depressive-like behaviors. We focused on WMI in this study because long-term neurological improvement was involved. Mice that underwent SIRPα-v Exo treatment showed superior nerve bundle integrity than those in the Con and NC Exo groups. Further TEM and CAP examinations revealed a definite protective effect of SIRPα-v Exos against myelin deficiency. Macroscopically and microscopically, SIRPα-v Exo treatment significantly reduced WMI in mice. We speculated that this effect was achieved by SIRPα-v Exos through the regulation of neuroinflammation. Unmodified NC Exos were also included in the experiment to exclude confounding factors. Consistent with the literature [54–56], NC Exos likewise exhibited therapeutic effects in some aspects, alleviating neurological dysfunction. MSC-exosomes not only have a delivery function but also therapeutic functions related to the unique properties of mesenchymal stem cells.

The function and status of microglia/macrophages change dynamically after ICH [13, 57–59]. Our data suggested a time-dependent expression of M1- and M2-phenotype markers and their associated cytokines. Given the ensuing secondary WMI, we inferred that anti-inflammatory processes are either insufficient or overwhelmed by the pro-inflammatory response in the natural course of ICH. When pro-inflammatory microglia/macrophages prevail, they trigger a cascade that starts secondary neuroinflammation around the hematoma, allowing necrosis to begin in nerve bundles that are merely displaced or compressed, ultimately causing irreversible WMI. By intervening in CD47-SIRPα signaling through SIRPα-v Exos, we succeeded in changing the trend. Blocking CD47-SIRPα signaling increased the proportion of M2 microglia/macrophages and decreased the proportion of M1 microglia/macrophages in striatal tissue surrounding the hematoma, supporting the notion that SIRPα-v Exos mitigated the early pro-inflammatory storm. However, it is undeniable that the SIRPα-v Exo treatment regimen does not completely reverse the immune response. Except for some long-term neurological functions, the protective effects of SIRPα-v Exos presented a statistical significance only in the first 7 days after ICH in most examinations. This could help us partially understand how SIRPα-v Exos modulate microglia/macrophages. More importantly, in subsequent experiments, we found that SIRPα-v Exos alone did not accomplish their regulatory functions in vitro. Simply, SIRPα-v Exo treatment has minimal regulatory effects on M2 microglia/macrophages in vitro, implying that we neglected some immune responses or cellular components involved in regulating neuroinflammation in vivo. The results also suggested that SIRPα-v Exos did not solely target microglia/macrophages for modulation but played a broader immunomodulatory role. Tregs have been suggested to prevent immunopathology caused by excessive immune responses [43]. Referring to recent investigations [44, 45, 60], we speculated that Tregs are likely to be involved, which were explored by flow cytometry. Our results revealed that in addition to directly suppressing M1 microglia/macrophages, SIRPα-v Exos recruited Tregs around the hematoma from peripheral blood and indirectly activated M2 microglia/macrophages. We attributed this extra immunomodulatory function of SIRPα-v Exos to the secretion of MSCs. Exosomes secreted by MSCs contain immunomodulatory factors that alter the expression of surface molecules and regulate T cells [61]. MSC-derived exosomes enhance Treg function to suppress immune responses and reduce inflammatory cytokines. Despite the fact that NC Exos recruited Tregs from peripheral blood after ICH, SIRPα-v Exos emerged a more significant effect. SIRPα variants play an equally critical role in the recruitment and regulation of Tregs, synergistically with intra-exosomal substances. As an integral part of the immune response, SIRPα-v Exos regulated the polarization of microglia/macrophages in concert with Tregs.

Previous studies have demonstrated that various pathways contribute to the activation and polarization of microglia/macrophages, such as the p38 MAPK-STAT1 and PI3K-Akt-mTOR signaling pathways [46, 47, 62, 63]. We further verified the mechanism of SIRPα-v Exos by in vitro experiments. Our results manifested that SIRPα-v Exos reduced neuroinflammation and prevented activated microglia from shifting to the M1-phenotype via inhibition of the p38 MAPK-STAT1 signaling pathway. Consistent with previous studies showing that MSC-derived exosomes regulated the M2 polarization of microglia/macrophages, our data suggested that modified exosomal SIRPα variants enhanced the regulatory effects of MSC-derived exosomes on M2 microglia. Moreover, SIRPα-v Exos recruited Tregs to coordinately regulate the polarization of microglia via the PI3K-Akt-mTOR signaling pathway.

It should be noted that to maintain the blood concentration of SIRPα-v Exos, we chose consecutive injections. This adds difficulties in pinpointing the time window for SIRPα-v Exo onset. Therefore, our current experimental design does not provide a good clarification of the mechanism of the protective effect against WMI. Whether SIRPα-v Exos provide a protective effect at an early stage or promote nerve fiber remyelination at a later stage requires further validation. Second, Tregs appear to play an essential role in the function of SIRPα-v Exos, but in fact, studies at this stage do not exactly clarify the target of SIRPα-v Exos. NC Exos contain substances that further enhance the function of SIRPα variants. However, both the efficacy and immunomodulatory function of NC Exos are inferior to those of SIRPα-v Exos, which may be the reason why stem cell exosomes have not been widely used for ICH treatment. It is unclear which substances in stem cell exosomes reinforce the regulatory effect of SIRPα variants on T cells. Further studies are necessary, and our own investigations are still ongoing. These efforts provide considerable grounds for the hope that this novel therapeutic approach could provide important clinical benefits for treating intracerebral hemorrhage and improving neurological functions.

## Conclusion

For the first time, we found that SIRPα-v Exos improved WMI and neurological dysfunction after ICH. SIRPα-v Exos regulated the polarization of microglia/macrophages. In addition, SIRPα-v Exos recruited Tregs to coordinately modulate the biofunction of microglia/macrophages. Collectively, our findings revealed that SIRPα-v Exos accelerated the clearance of hematoma and alleviated WMI by regulating the polarization of microglia/macrophages. The results of the present research can be significant for developing a novel therapeutic strategy for ICH.

## Supplementary Information

**Additional file 1: Figure S1.** Verification of the SIRPα variant. **Figure S2.** Construction and packaging of lentivirus. **Figure S3.** Lentiviral infection of MSCs. **Figure S4.** Analysis of membranous and cytoplasmic SIRPα variants in MSCs. **Figure S5.** NTA analysis of different batches of exosomes. **Figure S6.** Biodistribution of SIRPα-v Exos at 24 hours post injection. **Figure S7.** Representative immunofluorescence images of SIRPα-v Exos in brain tissue. **Figure S8.** Representative immunofluorescence images of released SIRPα-v. **Figure S9.** Representative immunofluorescence images of SIRPα-v Exos with microglia. **Figure S10A.** Serum biomarker assay after continuous SIRPα-v Exo administration. **Figure S11.** Representative histopathological images of hematoxylin and eosin‐ stained slides of major organs after continuous SIRPα-v Exo administration. **Figure S12.** Serum sTM and vWF assay after SIRPα-v Exo administration. **Figure S13.** Serial H.E. staining after ICH. **Figure S14.** Other typical results of the Morris water maze test. **Figure S15.** Long-term SIRPα-v Exo administration improves depressive-like behaviors after ICH. **Figure S16.** Culture and identification of primary microglia. **Figure S17.** Representative images of immunostaining of Tregs. **Table S1.** Hematological data obtained from the tail. **Table S2.** Antibodies, concentrations and manufacturers used. **Table S3.** The sequences of primers used for RT-qPCR.

## Data Availability

The datasets used and/or analyzed during the current study are available from the corresponding author on reasonable request.

## References

[1] Kellner CP, Song R, Troiani ZS, Ascanio LCMocco J.  (2020). Minimally invasive endoscopic evacuation of intracerebral haemorrhage: reaching the goal. Lancet.

[2] Asch C, Luitse MJ, Rinkel GJ, Tweel IKlijn CJ.  (2010). Incidence, case fatality, and functional outcome of intracerebral haemorrhage over time, according to age, sex, and ethnic origin: a systematic review and meta-analysis. Lancet Neurol.

[3] Xi G, Hua Y, Bhasin RR, Ennis SR, Keep RFHoff JT.  (2001). Mechanisms of edema formation after intracerebral hemorrhage: effects of extravasated red blood cells on blood flow and blood-brain barrier integrity. Stroke.

[4] Keep RF, Hua Y, Xi G (2012). Intracerebral haemorrhage: mechanisms of injury and therapeutic targets. Lancet Neurol..

[5] Dulamea AO (2017). The contribution of oligodendrocytes and oligodendrocyte progenitor cells to central nervous system repair in multiple sclerosis: perspectives for remyelination therapeutic strategies. Neural Regen Res.

[6] Chaudhary N, Pandey AS, Gemmete JJ, Hua Y, Huang Y, Gu Y (2015). Diffusion tensor imaging in hemorrhagic stroke. Exp Neurol.

[7] Zhao H, Pan P, Yang Y, Ge H, Chen W, Qu J (2017). Endogenous hydrogen sulphide attenuates NLRP3 inflammasome-mediated neuroinflammation by suppressing the P2X7 receptor after intracerebral haemorrhage in rats. J Neuroinflammation.

[8] Miron VE, Franklin RJ (2014). Macrophages and CNS remyelination. J Neurochem..

[9] Larochelle A, Bellavance M-A, Michaud J-P, Rivest S (2016). Bone marrow-derived macrophages and the CNS: an update on the use of experimental chimeric mouse models and bone marrow transplantation in neurological disorders. Biochim Biophys Acta BBA Mol Basis Dis..

[10] Chhor V, Le Charpentier T, Lebon S, Oré M-V, Celador IL, Josserand J (2013). Characterization of phenotype markers and neuronotoxic potential of polarised primary microglia in vitro. Brain Behav Immun.

[11] Martinez FO, Helming L, Gordon S (2009). Alternative activation of macrophages: an immunologic functional perspective. Annu Rev Immunol..

[12] Hu X, Leak RK, Shi Y, Suenaga J, Gao Y, Zheng P (2015). Microglial and macrophage polarization—new prospects for brain repair. Nat Rev Neurol.

[13] Yang J, Ding S, Huang W, Hu J, Huang S, Zhang Y (2016). Interleukin-4 ameliorates the functional recovery of intracerebral hemorrhage through the alternative activation of microglia/macrophage. Front Neurosci.

[14] Zhao H, Garton T, Keep RF, Hua Y, Xi G (2015). Microglia/Macrophage Polarization After Experimental Intracerebral Hemorrhage. Transl Stroke Res..

[15] Ni W, Mao S, Xi G, Keep RF, Hua Y (2016). Role of Erythrocyte CD47 in Intracerebral Hematoma Clearance. Stroke..

[16] Matozaki T, Murata Y, Okazawa H, Ohnishi H (2009). Functions and molecular mechanisms of the CD47-SIRPalpha signalling pathway. Trends Cell Biol..

[17] van den Berg TK, van Beek EM, Bühring HJ, Colonna M, Hamaguchi M, Howard CJ (2005). A nomenclature for signal regulatory protein family members. J Immunol.

[18] Kharitonenkov A, Chen Z, Sures I, Wang H, Schilling J, Ullrich A (1997). A family of proteins that inhibit signalling through tyrosine kinase receptors. Nature..

[19] Han MH, Lundgren DH, Jaiswal S, Chao M, Graham KL, Garris CS (2012). Janus-like opposing roles of CD47 in autoimmune brain inflammation in humans and mice. J Exp Med.

[20] Cheng L, Zhang X, Tang J, Lv Q, Liu J (2021). Gene-engineered exosomes-thermosensitive liposomes hybrid nanovesicles by the blockade of CD47 signal for combined photothermal therapy and cancer immunotherapy. Biomaterials..

[21] Pan Y, Wang F, Liu Y, Jiang J, Yang YG, Wang H (2014). Studying the mechanism of CD47-SIRPα interactions on red blood cells by single molecule force spectroscopy. Nanoscale..

[22] Majeti R, Chao MP, Alizadeh AA, Pang WW, Jaiswal S, Gibbs KD (2009). CD47 is an adverse prognostic factor and therapeutic antibody target on human acute myeloid leukemia stem cells. Cell.

[23] Chao MP, Alizadeh AA, Tang C, Myklebust JH, Varghese B, Gill S (2010). Anti-CD47 antibody synergizes with rituximab to promote phagocytosis and eradicate non-Hodgkin lymphoma. Cell.

[24] Weiskopf K, Ring AM, Ho CC, Volkmer JP, Levin AM, Volkmer AK (2013). Engineered SIRPα variants as immunotherapeutic adjuvants to anticancer antibodies. Science.

[25] Le Blanc K, Davies LC (2015). Mesenchymal stromal cells and the innate immune response. Immunol Lett..

[26] Forbes GM, Sturm MJ, Leong RW, Sparrow MP, Segarajasingam D, Cummins AG (2014). A phase 2 study of allogeneic mesenchymal stromal cells for luminal Crohn’s disease refractory to biologic therapy. Clin Gastroenterol Hepatol.

[27] Moskowitz MA, Lo EH, Iadecola C (2010). The science of stroke: mechanisms in search of treatments. Neuron..

[28] Zhang ZG, Chopp M (2009). Neurorestorative therapies for stroke: underlying mechanisms and translation to the clinic. Lancet Neurol..

[29] Yagi H, Soto-Gutierrez A, Parekkadan B, Kitagawa Y, Tompkins RG, Kobayashi N (2010). Mesenchymal stem cells: Mechanisms of immunomodulation and homing. Cell Transplant.

[30] Théry C, Zitvogel L, Amigorena S (2002). Exosomes: composition, biogenesis and function. Nat Rev Immunol..

[31] Xin H, Katakowski M, Wang F, Qian J-Y, Liu XS, Ali MM (2017). MicroRNA-17–92 cluster in exosomes enhance neuroplasticity and functional recovery after stroke in rats. Stroke.

[32] Cui GH, Guo HD, Li H, Zhai Y, Gong ZB, Wu J (2019). RVG-modified exosomes derived from mesenchymal stem cells rescue memory deficits by regulating inflammatory responses in a mouse model of Alzheimer’s disease. Immun Ageing.

[33] Kim DK, Nishida H, An SY, Shetty AK, Bartosh TJ, Prockop DJ (2016). Chromatographically isolated CD63+CD81+ extracellular vesicles from mesenchymal stromal cells rescue cognitive impairments after TBI. Proc Natl Acad Sci U S A..

[34] Duan S, Wang F, Cao J, Wang C (2020). Exosomes Derived from MicroRNA-146a-5p-Enriched Bone Marrow Mesenchymal Stem Cells Alleviate Intracerebral Hemorrhage by Inhibiting Neuronal Apoptosis and Microglial M1 Polarization. Drug Des Devel Ther..

[35] Koh E, Lee EJ, Nam GH, Hong Y, Cho E, Yang Y (2017). Exosome-SIRPα, a CD47 blockade increases cancer cell phagocytosis. Biomaterials.

[36] Yang H, Ni W, Wei P, Li S, Gao X, Su J (2021). HDAC inhibition reduces white matter injury after intracerebral hemorrhage. J Cereb Blood Flow Metab.

[37] Abello J, Nguyen TDT, Marasini R, Aryal S, Weiss ML (2019). Biodistribution of gadolinium- and near infrared-labeled human umbilical cord mesenchymal stromal cell-derived exosomes in tumor bearing mice. Theranostics..

[38] Fenalti G, Villanueva N, Griffith M, Pagarigan B, Lakkaraju SK, Huang RY (2021). Structure of the human marker of self 5-transmembrane receptor CD47. Nat Commun.

[39] Theocharides AP, Jin L, Cheng PY, Prasolava TK, Malko AV, Ho JM (2012). Disruption of SIRPα signaling in macrophages eliminates human acute myeloid leukemia stem cells in xenografts. J Exp Med.

[40] Willingham SB, Volkmer JP, Gentles AJ, Sahoo D, Dalerba P, Mitra SS (2012). The CD47-signal regulatory protein alpha (SIRPa) interaction is a therapeutic target for human solid tumors. Proc Natl Acad Sci U S A.

[41] Klugah-Brown B, Wang P, Jiang Y, Becker B, Hu P, Uddin LQ, et al. Structural-functional connectivity mapping of the insular cortex: a combined data-driven and meta-analytic topic mapping. Cereb Cortex. 2022;bhac168. 10.1093/cercor/bhac168 35511500

[42] Zhang Q, Zhu W, Xu F, Dai X, Shi L, Cai W (2019). The interleukin-4/PPARγ signaling axis promotes oligodendrocyte differentiation and remyelination after brain injury. PLoS Biol.

[43] Machhi J, Kevadiya BD, Muhammad IK, Herskovitz J, Olson KE, Mosley RL (2020). Harnessing regulatory T cell neuroprotective activities for treatment of neurodegenerative disorders. Mol Neurodegener.

[44] Machhi J, Yeapuri P, Lu Y, Foster E, Chikhale R, Herskovitz J (2021). CD4+ effector T cells accelerate Alzheimer’s disease in mice. J Neuroinflammation.

[45] Shi L, Sun Z, Su W, Xu F, Xie D, Zhang Q (2021). Treg cell-derived osteopontin promotes microglia-mediated white matter repair after ischemic stroke. Immunity.

[46] Swaroop S, Sengupta N, Suryawanshi AR, Adlakha YK, Basu A (2016). HSP60 plays a regulatory role in IL-1β-induced microglial inflammation via TLR4-p38 MAPK axis. J Neuroinflammation..

[47] Fang Y, Jiang Q, Li S, Zhu H, Xu R, Song N (2021). Opposing functions of β-arrestin 1 and 2 in Parkinson’s disease via microglia inflammation and Nprl3. Cell Death Differ.

[48] Wang J, Doré S (2007). Heme oxygenase-1 exacerbates early brain injury after intracerebral haemorrhage. Brain..

[49] Wang J, Dore S (2008). Heme oxygenase 2 deficiency increases brain swelling and inflammation after intracerebral hemorrhage. Neuroscience..

[50] Savill J, Fadok V (2000). Corpse clearance defines the meaning of cell death. Nature..

[51] Edris B, Weiskopf K, Volkmer AK, Volkmer JP, Willingham SB, Contreras-Trujillo H (2012). Antibody therapy targeting the CD47 protein is effective in a model of aggressive metastatic leiomyosarcoma. Proc Natl Acad Sci U S A.

[52] Beckman RA, Weiner LM, Davis HM (2007). Antibody constructs in cancer therapy: protein engineering strategies to improve exposure in solid tumors. Cancer..

[53] Tabrizi MA, Roskos LK (2007). Preclinical and clinical safety of monoclonal antibodies. Drug Discov Today..

[54] Otero-Ortega L, Gómez de Frutos MC, Laso-García F, Rodríguez-Frutos B, Medina-Gutiérrez E, Lopez JA (2018). Exosomes promote restoration after an experimental animal model of intracerebral hemorrhage. J Cereb Blood Flow Metab..

[55] Han Y, Seyfried D, Meng Y, Yang D, Schultz L, Chopp M (2018). Multipotent mesenchymal stromal cell-derived exosomes improve functional recovery after experimental intracerebral hemorrhage in the rat. J Neurosurg.

[56] Bedini G, Bersano A, Zanier ER, Pischiutta F, Parati EA (2018). Mesenchymal Stem Cell Therapy in Intracerebral Haemorrhagic Stroke. Curr Med Chem..

[57] Kroner A, Greenhalgh AD, Zarruk JG, Dos Santos RP, Gaestel M, David S (2014). TNF and increased intracellular iron alter macrophage polarization to a detrimental M1 phenotype in the injured spinal cord. Neuron..

[58] Hayakawa K, Okazaki R, Morioka K, Nakamura K, Tanaka S, Ogata T (2014). Lipopolysaccharide preconditioning facilitates M2 activation of resident microglia after spinal cord injury. J Neurosci Res..

[59] Li M, Li Z, Ren H, Jin WN, Wood K, Liu Q (2017). Colony stimulating factor 1 receptor inhibition eliminates microglia and attenuates brain injury after intracerebral hemorrhage. J Cereb Blood Flow Metab.

[60] Liesz A, Suri-Payer E, Veltkamp C, Doerr H, Sommer C, Rivest S (2009). Regulatory T cells are key cerebroprotective immunomodulators in acute experimental stroke. Nat Med.

[61] Shi Y, Su J, Roberts AI, Shou P, Rabson ABRen G.  (2012). How mesenchymal stem cells interact with tissue immune responses. Trends Immunol.

[62] Gaojian T, Dingfei Q, Linwei L, Xiaowei W, Zheng Z, Wei L (2020). Parthenolide promotes the repair of spinal cord injury by modulating M1/M2 polarization via the NF-κB and STAT 1/3 signaling pathway. Cell Death Discov.

[63] Rosborough BR, Raïch-Regué D, Matta BM, Lee K, Gan B, DePinho RA (2013). Murine dendritic cell rapamycin-resistant and rictor-independent mTOR controls IL-10, B7–H1, and regulatory T-cell induction. Blood.

