# Supplementary material for: Modified exosomal SIRPα variants alleviate white matter injury after intracerebral hemorrhage via microglia/macrophages
Source: Biomater Res. 2022 Nov 26;26:67. doi: 10.1186/s40824-022-00311-4 (PMC9701394; doi:10.1186/s40824-022-00311-4)
Supplement: Supplementary file 1 — Additional file 1: Figure S1. Verification of the SIRPα variant. Figure S2. Construction and packaging of lentivirus. Figure S3. Lentiviral infection of MSCs. Figure S4. Analysis of membranous and cytoplasmic SIRPα variants in MSCs. Figure S5. NTA analysis of different batches of exosomes. Figure S6. Biodistribution of SIRPα-v Exos at 24 hours post injection. Figure S7. Representative immunofluorescence images of SIRPα-v Exos in brain tissue. Figure S8. Representative immunofluorescence images of released SIRPα-v. Figure S9. Representative immunofluorescence images of SIRPα-v Exos with microglia. Figure S10A. Serum biomarker assay after continuous SIRPα-v Exo administration. Figure S11. Representative histopathological images of hematoxylin and eosin‐ stained slides of major organs after continuous SIRPα-v Exo administration. Figure S12. Serum sTM and vWF assay after SIRPα-v Exo administration. Figure S13. Serial H.E. staining after ICH. Figure S14. Other typical results of the Morris water maze test. Figure S15. Long-term SIRPα-v Exo administration improves depressive-like behaviors after ICH. Figure S16. Culture and identification of primary microglia. Figure S17. Representative images of immunostaining of Tregs. Table S1. Hematological data obtained from the tail. Table S2. Antibodies, concentrations and manufacturers used. Table S3. The sequences of primers used for RT-qPCR. [file 40824_2022_311_MOESM1_ESM.pdf]

Supplementary Information

Modified exosomal SIRPα variants alleviate white matter injury after intracerebral hemorrhage via microglia/macrophages

Xinjie Gao[1†], Heng Yang[1, 4†], Weiping Xiao[1], Jiabin Su[1, 3], Yuwen Zhang[5], He Wang[5*], Wei Ni[1, 2*], Yuxiang Gu[1, 2*]

1. Department of Neurosurgery, Huashan Hospital, Fudan University, Shanghai 200040, China
2. National Center for Neurological Disorders, Shanghai 201107, China
3. Neurosurgical Institute of Fudan University, Shanghai 201107, China
4. Shanghai Key Laboratory of Brain Function and Restoration and Neural Regeneration, Shanghai 200052, China
5. Institute of Science and Technology for Brain-inspired Intelligence, Fudan University, Shanghai 200433, China

[†] Xinjie Gao and Heng Yang contributed equally to this work.

* E-mail: hewang@fudan.edu.cn (He Wang); hsniwei@fudan.edu.cn (Wei Ni); guyuxiang1972@126.com (Yuxiang Gu)

Figure S1.

A

EEE**V**Q**I**IQPDKSVLVAAGETATLRCT**I**TSL**F**PVGPIQWFRGAGPGR**V**LIYNQ**RQ**G**P**FPRVTT**I**SD
TT**R**RNNMDFSIRIGNITPADAGTYYC**I**KFRKGSPDDVEFKSGAGTELSVRAKPSAPVVSGPAA

B

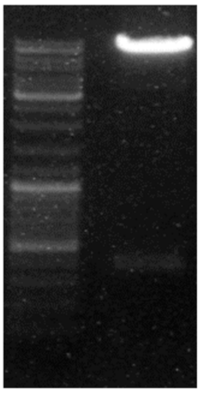

C

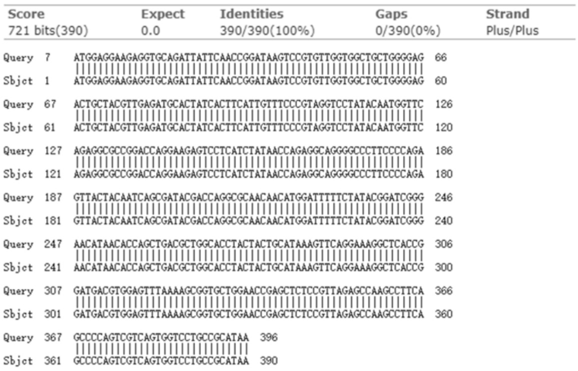

Figure S1. Verification of the SIRPα variant. A De novo sequencing of SIRPα variant (V5) in exosomes by mass spectrometry. The complete amino acid sequences were analyzed and assembled by PEAKS AB. The red and bold letters are the abbreviated names of mutated amino acids. **B** The result of restriction enzyme detection in the pCDH-CMV-MCS-EF1-copGFP lentiviral vector. **C** The results of codon sequencing of the SIRPα variant in lentivirus. *Query* represents the actual sequence, and *Sbjct* represents the designed sequence.

Figure S2.

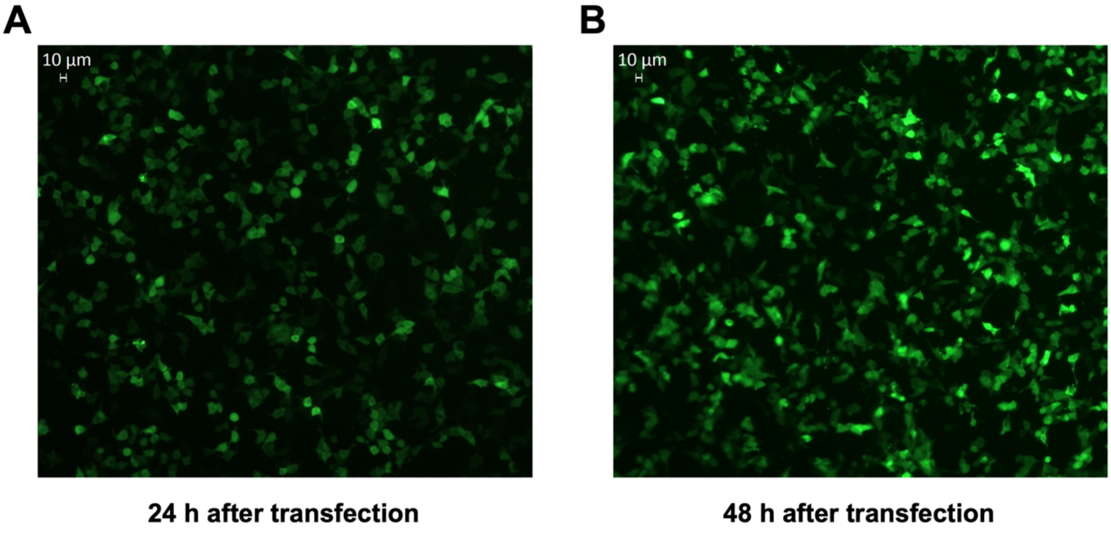

Figure S2. Construction and packaging of lentivirus. A Representative image of 293T cells at 24 hours after transfection of plasmid loading the sequence of engineered SIRPα variant. The 293T cells, presenting green fluorescence, began to express sequences on the plasmid (bar = 10 µm). **B** Representative image of 293T cells at 48 hours after transfection of plasmid with a stronger green fluorescence because of the expression of green fluorescent protein (GFP) (bar = 10 µm).

Figure S3.

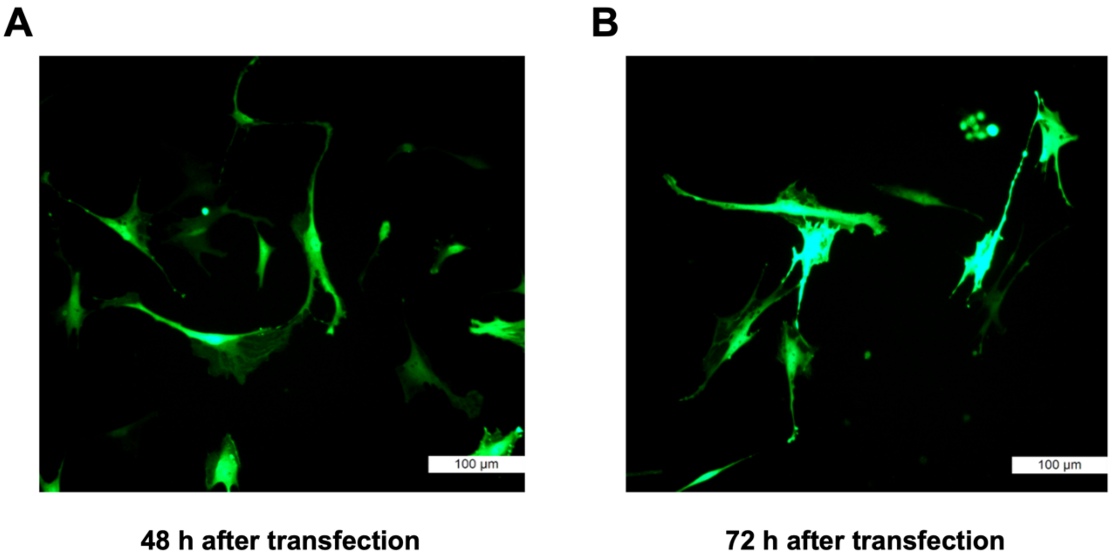

Figure S3. Lentiviral infection of MSCs. A Representative image of MSCs 48 hours after lentiviral infection. The green fluorescence indicated that BMSCs began to express the sequence of the engineered SIRPα variant (bar = 100 μm). **B** Representative image of MSCs with stronger green fluorescence 72 hours after lentiviral infection (bar = 100 μm).

Figure S4.

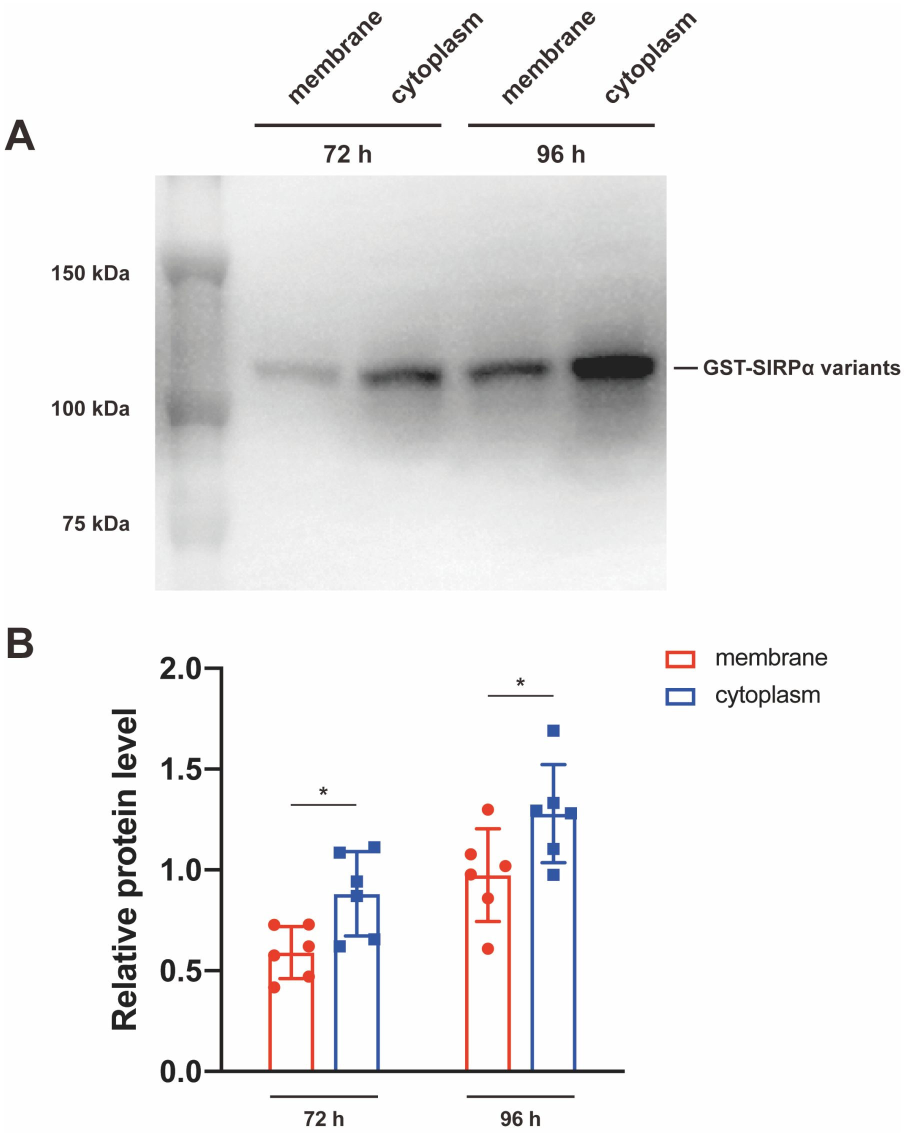

Figure S4. Analysis of membranous and cytoplasmic SIRPα variants in MSCs. A Western blot images showing the protein levels of membranous and cytoplasmic GST-SIRPα variants in MSCs 72 hours and 96 hours post-transfection. **B** Quantification analysis of membranous and cytoplasmic expression of GST-SIRPα variants (n = 6/group). * P<0.05. All the data are presented as the mean ± SD.

Figure S5.

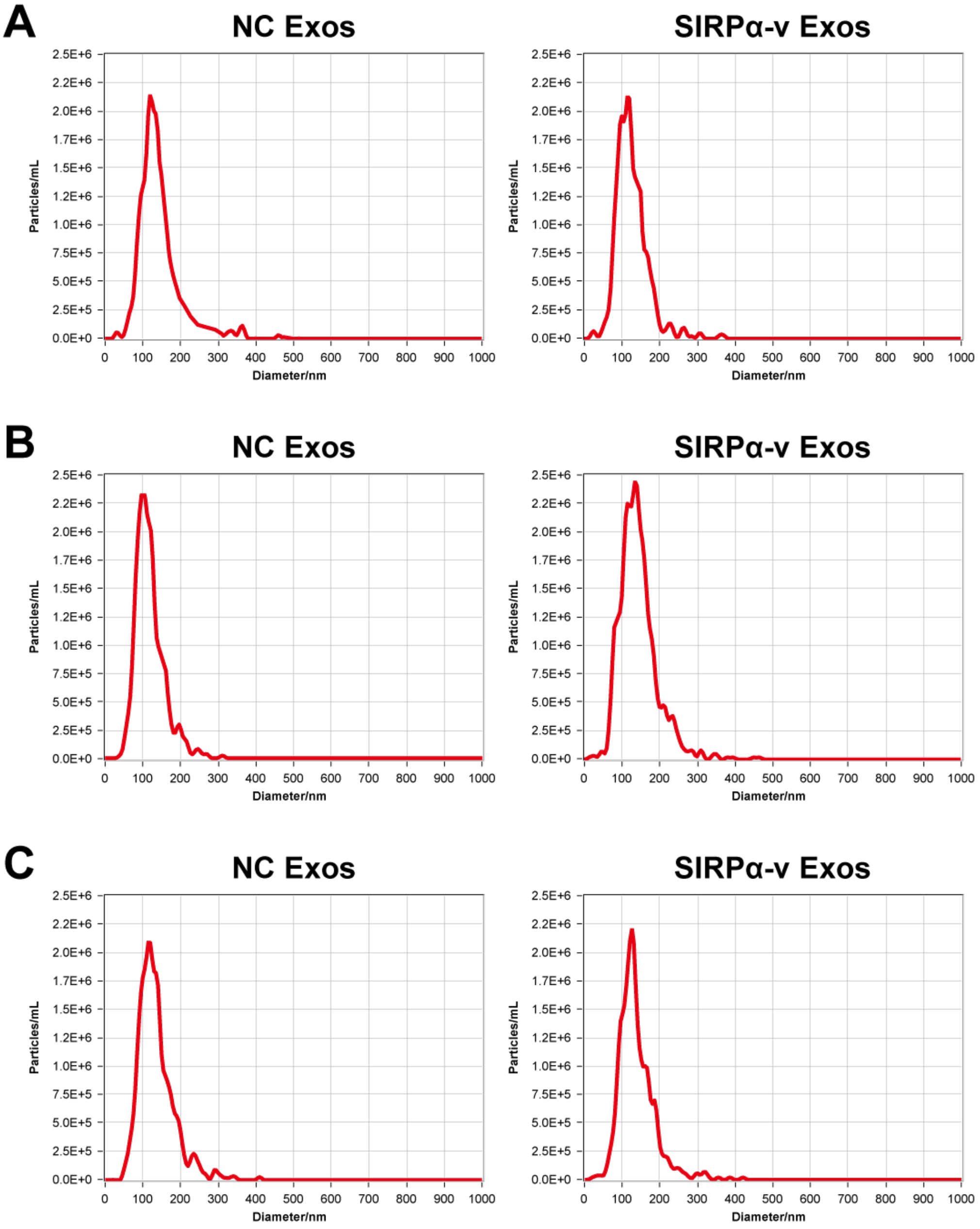

Figure S5. NTA analysis of different batches of exosomes. A-C The fitting curves of different batches of exosomes for NTA analysis **A** Stored NC and SIRPα-v exosome samples in the mid-stage of the study. **B** Stored NC and SIRPα-v exosome samples in the late stage of the study. **C** NC and SIRPα-v exosomes extracted for revision experiments.

Figure S6.

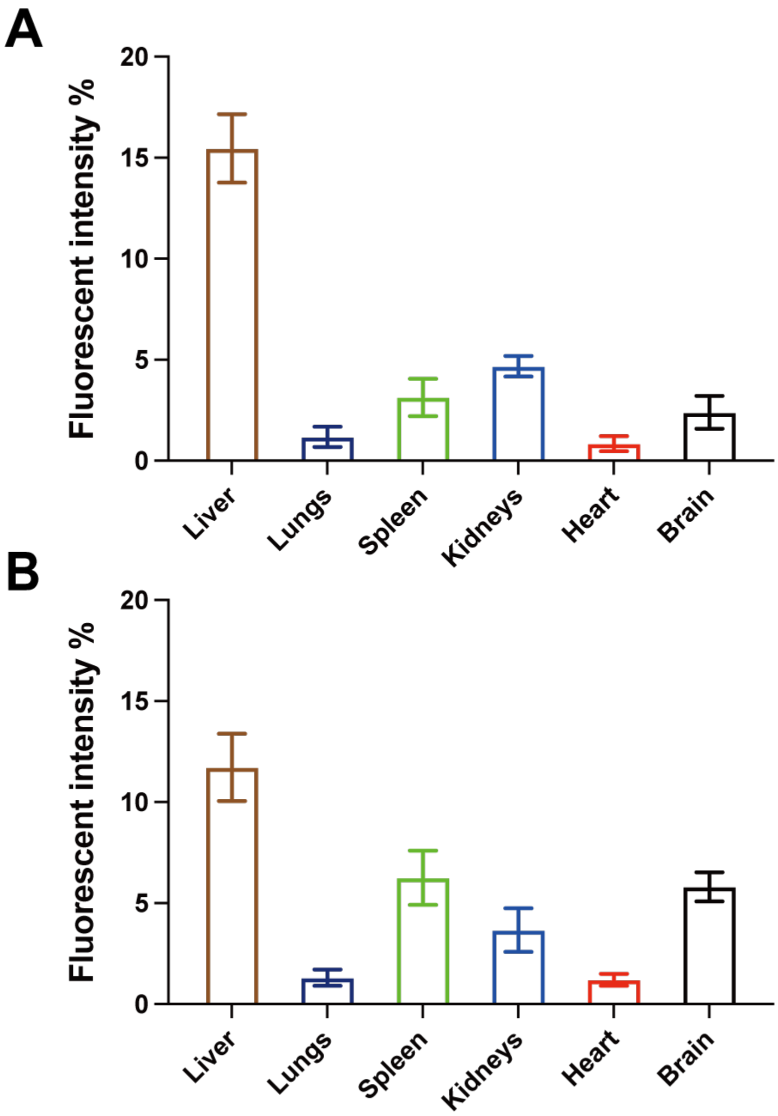

Figure S6. Biodistribution of SIRPα-v Exos at 24 hours post injection. After the 24 h survival period, the organs were collected, and the fluorescence intensity was individually assessed. **A** Quantitative analysis of the fluorescence intensity of SIRPα-v Exos in normal mice. **B** Quantitative analysis of the fluorescence intensity of SIRPα-v Exos in mice suffering from ICH. The graph shows the signal intensity per gram of tissue in the main organs. All the data are presented as the mean ± SD, n = 5.

Figure S7.

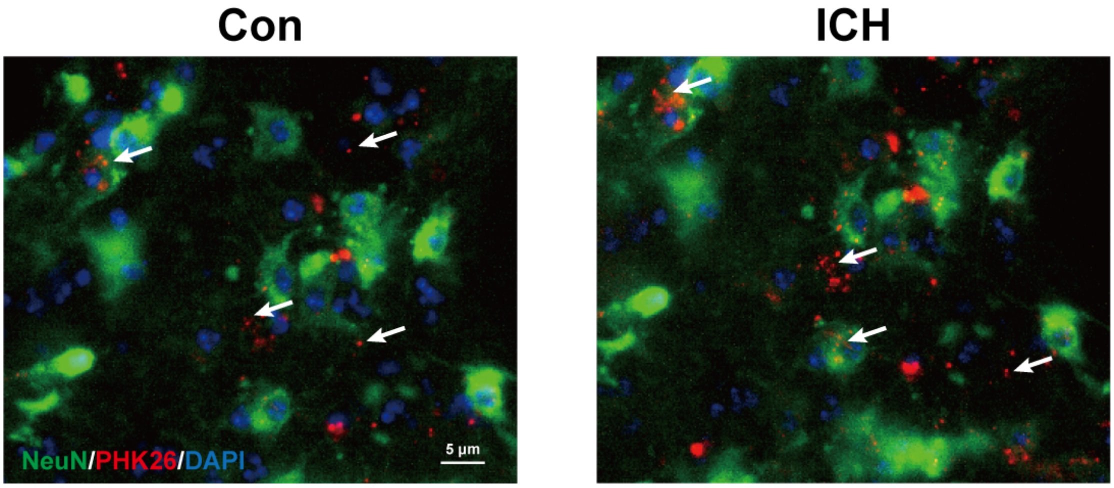

Figure S7. Representative immunofluorescence images of SIRPα-v Exos in brain tissue. PKH26-labeled SIRPα-v Exos could be observed by immunofluorescence (red) in the brain tissue (green and blue) of normal and ICH mice 24 hours after intravenous administration. SIRPα-v Exos in the perihematomal region of the brain appeared to be increased in mice suffering from ICH compared to normal mice.

Figure S8.

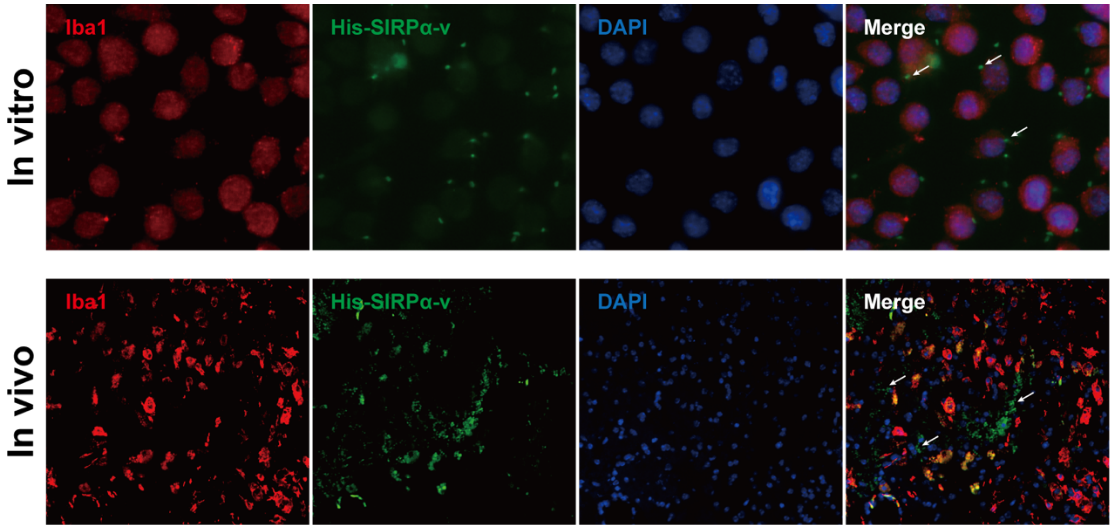

Figure S8. Representative immunofluorescence images of released SIRPα-v. SIRPα-v released from exosomes carries a His-tag. Erythrocytes bound to released SIRPα-v could be labeled by His-tag. ***In vitro.*** Exosomes were added to microglia (red) and erythrocytes under coculture conditions. Released SIRPα-v was detected 24 hours after administration. Erythrocytes bound to SIRPα-v (green) could be observed. ***In vivo.*** Erythrocytes bound to SIRPα-v (green) were observed in hematomas that were not separated from brain tissue 24 hours after SIRPα-v Exo injection intravenously. A small number of microglia could be colabeled with His-tag (green) and Iba1 (red). The white arrows indicate erythrocytes bound to released SIRPα-v.

Figure S9.

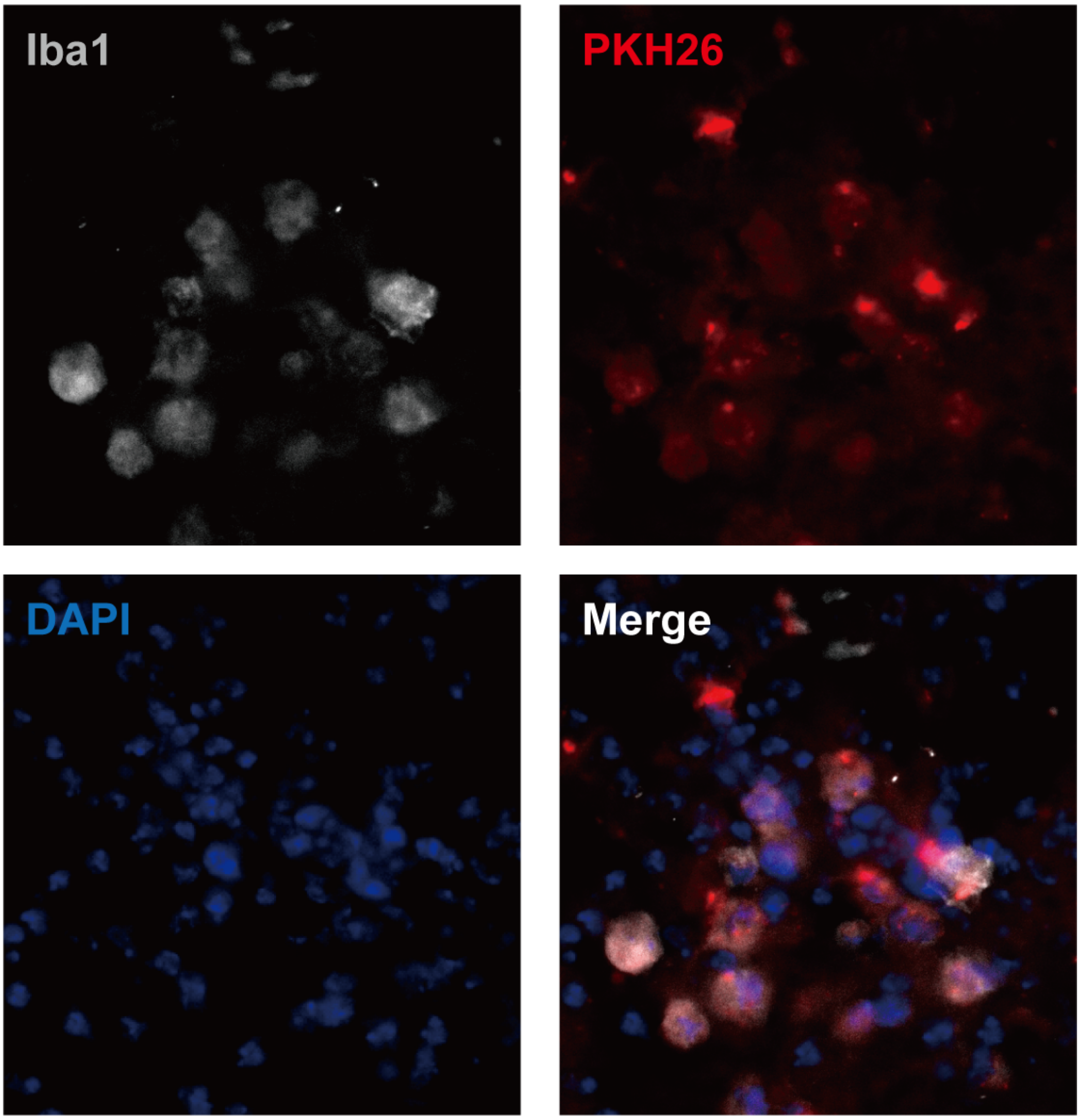

Figure S9. Representative immunofluorescence images of SIRPα-v Exos with microglia. SIRPα-v Exos were labeled with PKH26 in advance. Twenty-four hours after SIRPα-v Exos were injected intravenously, SIRPα-v Exos (red) and microglia (gray) were copresented, and some microglia were labeled with PKH26, indicating the interaction between SIRPα-v Exos and microglia.

Figure S10.

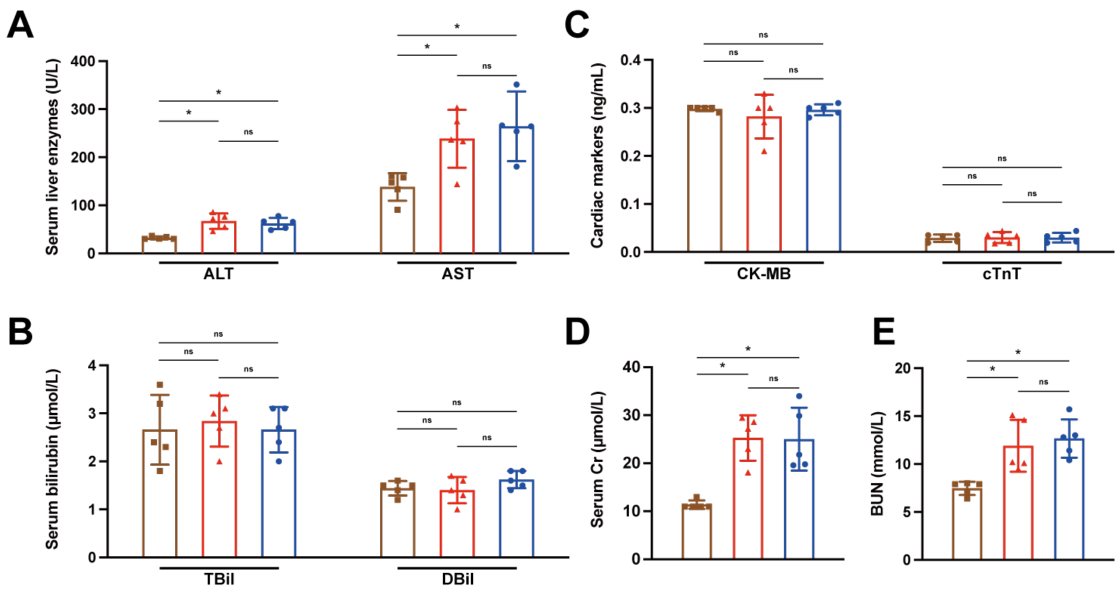

Figure S10A. Serum biomarker assay after continuous SIRPα-v Exo administration. SIRPα-v Exos accumulate in organs other than the brain, such as the heart, liver, lungs and kidneys. Serum biomarkers respond to organ damage. Liver enzymes and bilirubin reflect liver injury. CK-MB and cTnT reflect myocardial injury. Cr and BUN reflect kidney injury. Serum biomarkers were examined in mice 14 days after continuous injection. **A-B** Continuous administration of both SIRPα-v Exos and anti-CD47 antibodies caused mild elevations in ALT and AST, suggesting liver injury. No significant alterations in bilirubin were detected. **C** Neither SIRPα-v Exos nor anti-CD47 antibodies caused elevations in biomarkers of myocardial injury. **D-E** Continuous administration of SIRPα-v Exos and anti-CD47 antibodies resulted in elevated Cr and BUN, suggesting renal injury. n = 5/group. * P<0.05, no significance. All the data are presented as the mean ± SD.

Figure S11.

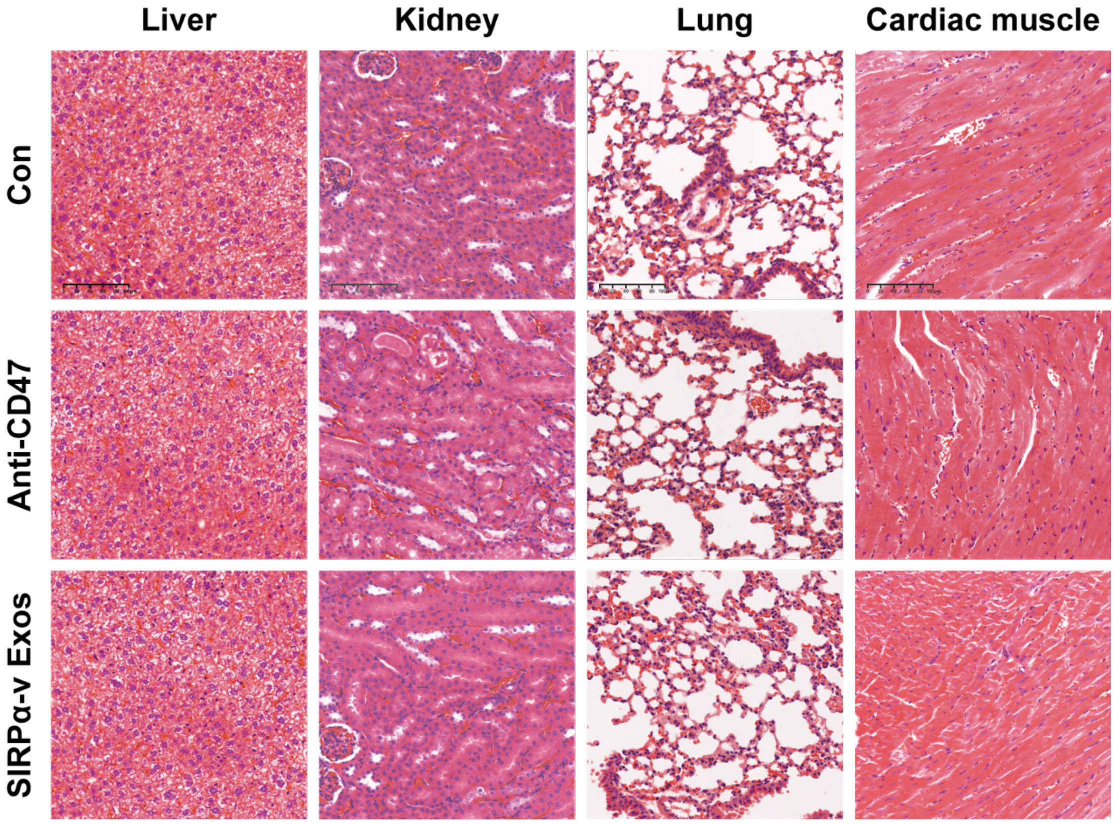

Figure S11. Representative histopathological images of hematoxylin and eosin-stained slides of major organs after continuous SIRPα-v Exo administration. ***Liver.*** The slides showed no hepatocyte damage, inflammation or necrosis, little immune cell infiltrated, and an orderly arrangement of liver sinusoidal structures. ***Kidney.*** No tubular dilation, brush border loss, nuclear loss or cast formation were observed in kidney slides. ***Lung.*** No capillary dilation and no exudate in the alveoli were observed in the lung slides. There was no significant infiltration of inflammatory cells between alveoli. ***Heart.*** Myocardial fibers were clear. No necrosis of cardiomyocytes or cardiomyocyte lysis was observed.

Figure S12.

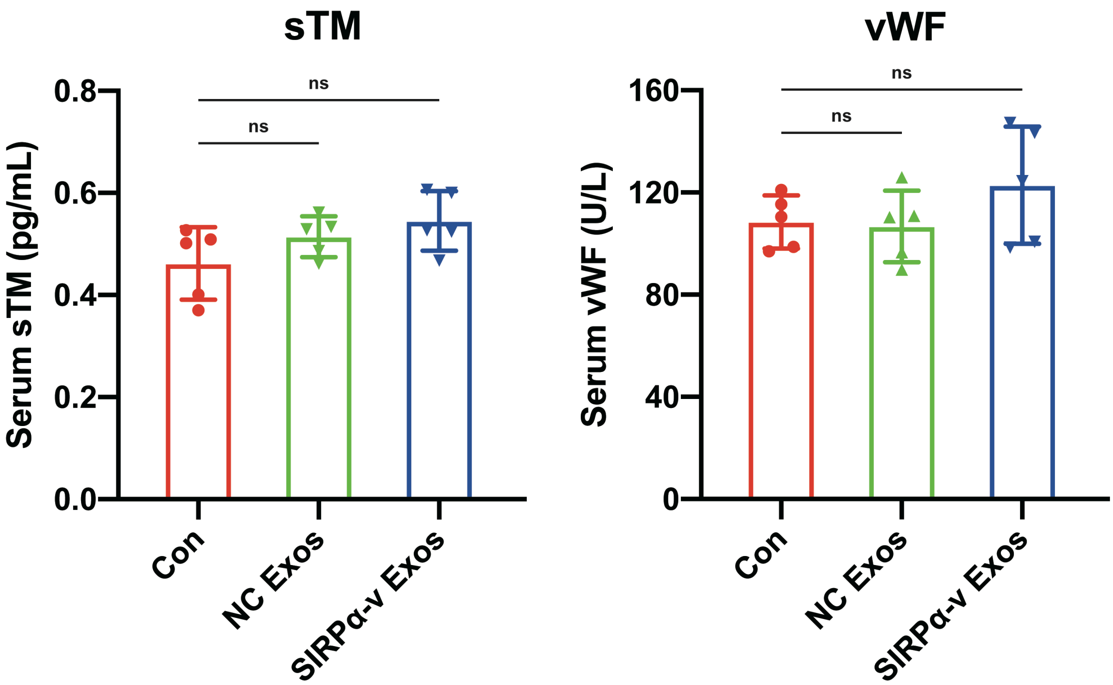

Figure S12. Serum sTM and vWF assay after SIRPα-v Exo administration.

Serum sTM and vWF are biomarkers of vascular endothelial injury. We analyzed serum sTM and vWF by ELISA after 3 days of continuous SIRPα-v Exo administration. Neither NC Exos nor SIRPα-v Exos resulted in significant vascular endothelial injuries (n = 5/group). ns, no significance. All the data are presented as the mean ± SD.

Figure S13.

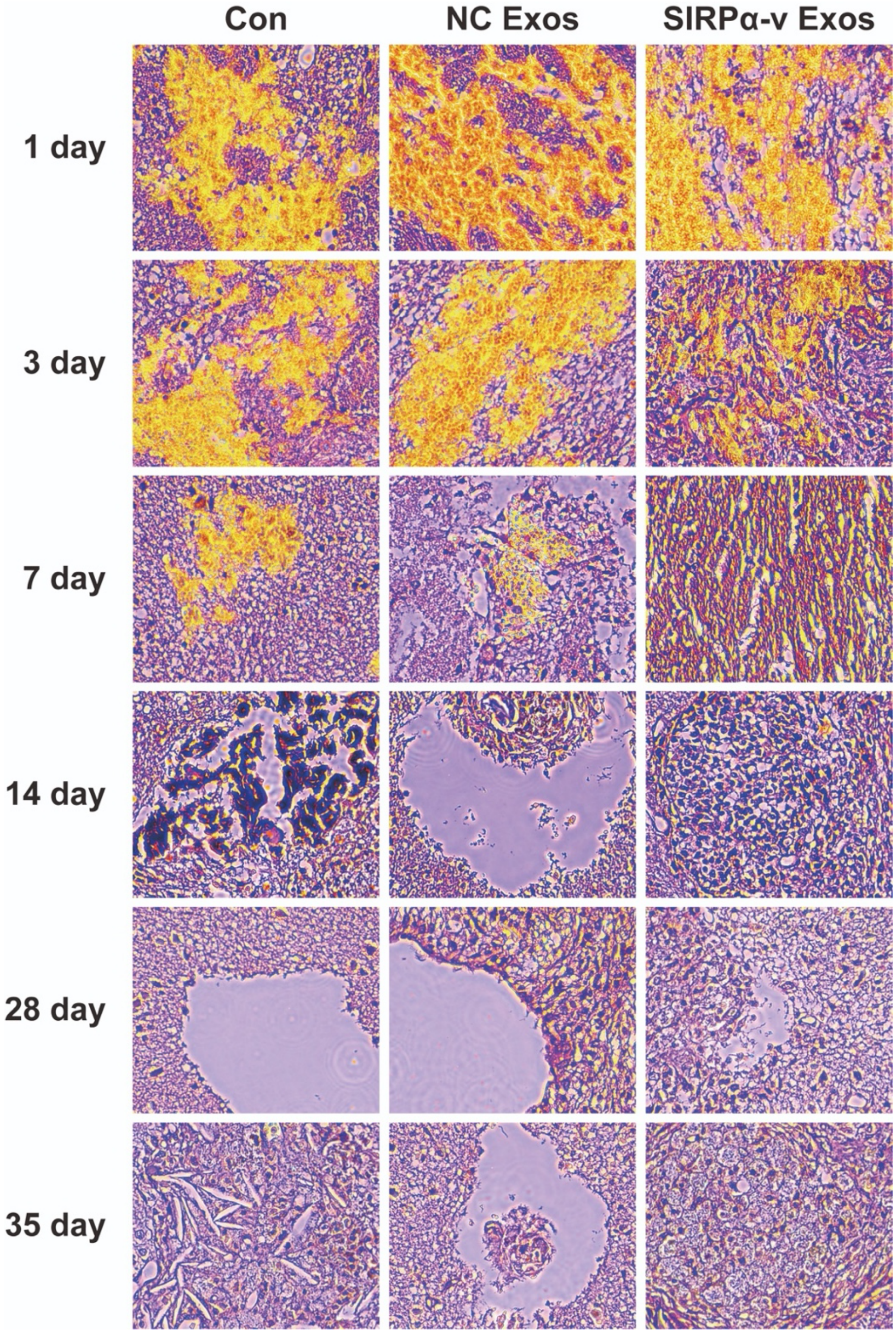

Figure S13. Serial H.E. staining after ICH.

The HE staining results suggested that SIRPα-v Exos accelerated the clearance of the hematoma. The mice in the SIRPα-v Exo group had almost complete removal of the hematoma on the 7th day after ICH, faster than those in the Con and NC Exo groups.

Figure S14.

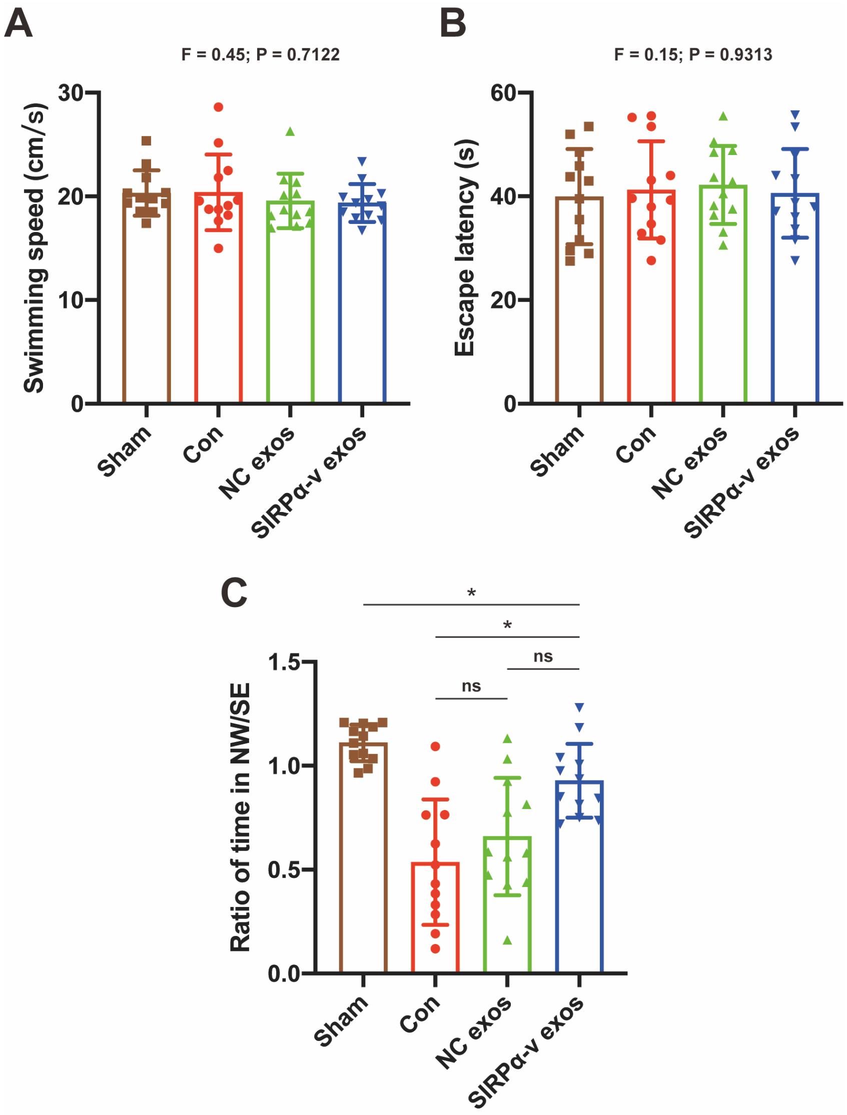

Figure S14. Other typical results of the Morris water maze test. A Quantitative analysis of the swimming speed of mice among the different groups in the visible platform trial. **B** Quantitative analysis of the escape latency of mice among the different groups in the visible platform trial. **C** The ratio of the time spent in the quadrant of the platform (NW quadrant) to the time spent in the diagonal quadrant of the platform (SE quadrant). n = 12/group, * P<0.05, ns, no significance. All the data are presented as the mean ± SD.

Figure S15.

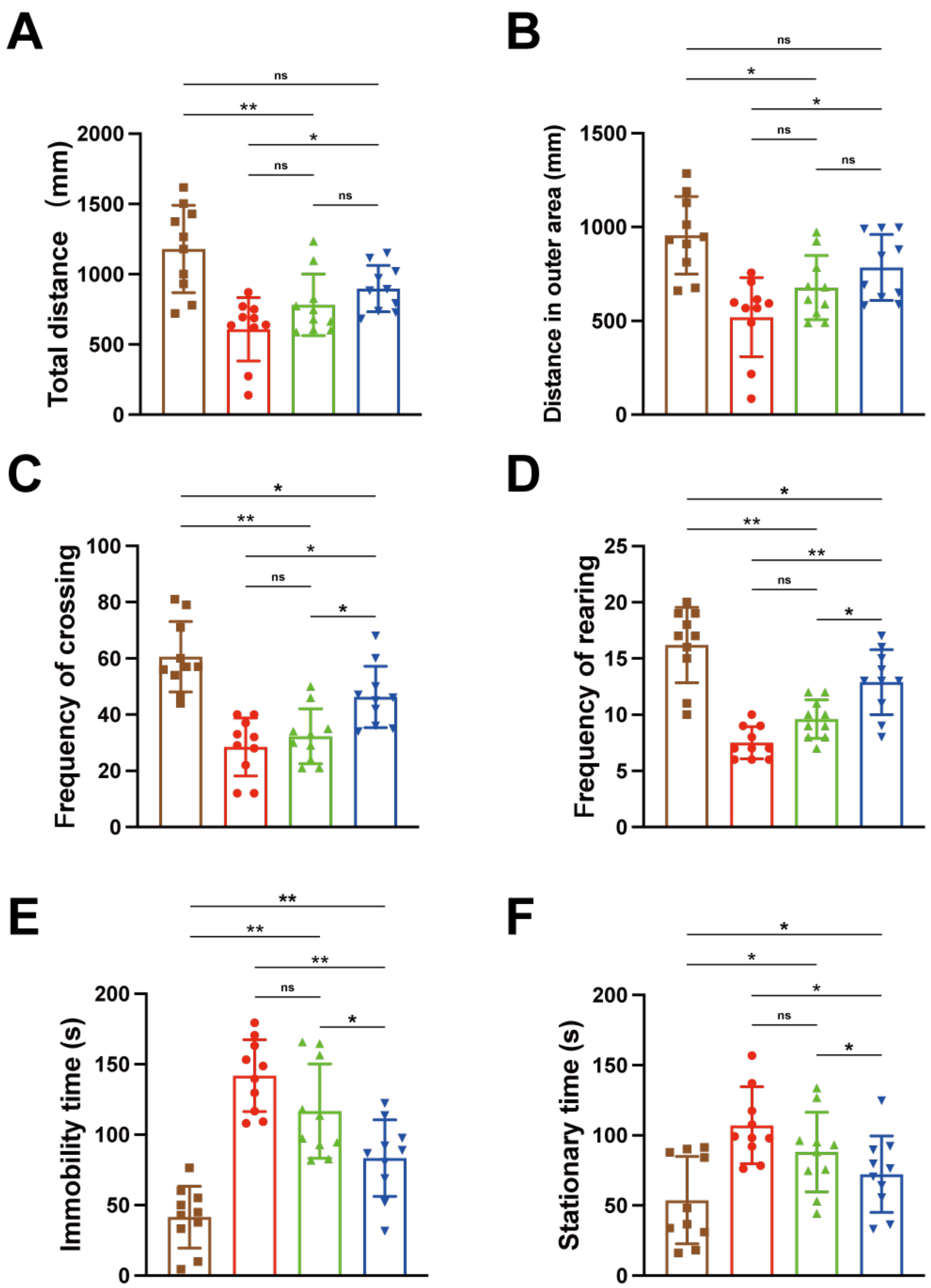

Figure S15. Long-term SIRPα-v Exo administration improves depressive-like behaviors after ICH. A-D Open-field test: A The total distance traveled by mice in the open field. B Time spent in the outer area. C The frequency of grid-crossing. D The frequency of rearing. **E** Duration of immobility in the forced swim test. **F** Duration of immobility in the tail suspension test. n = 10/group. * P<0.05, ** P<0.01, ns, no significance. All the data are presented as the mean ± SD.

Figure S16

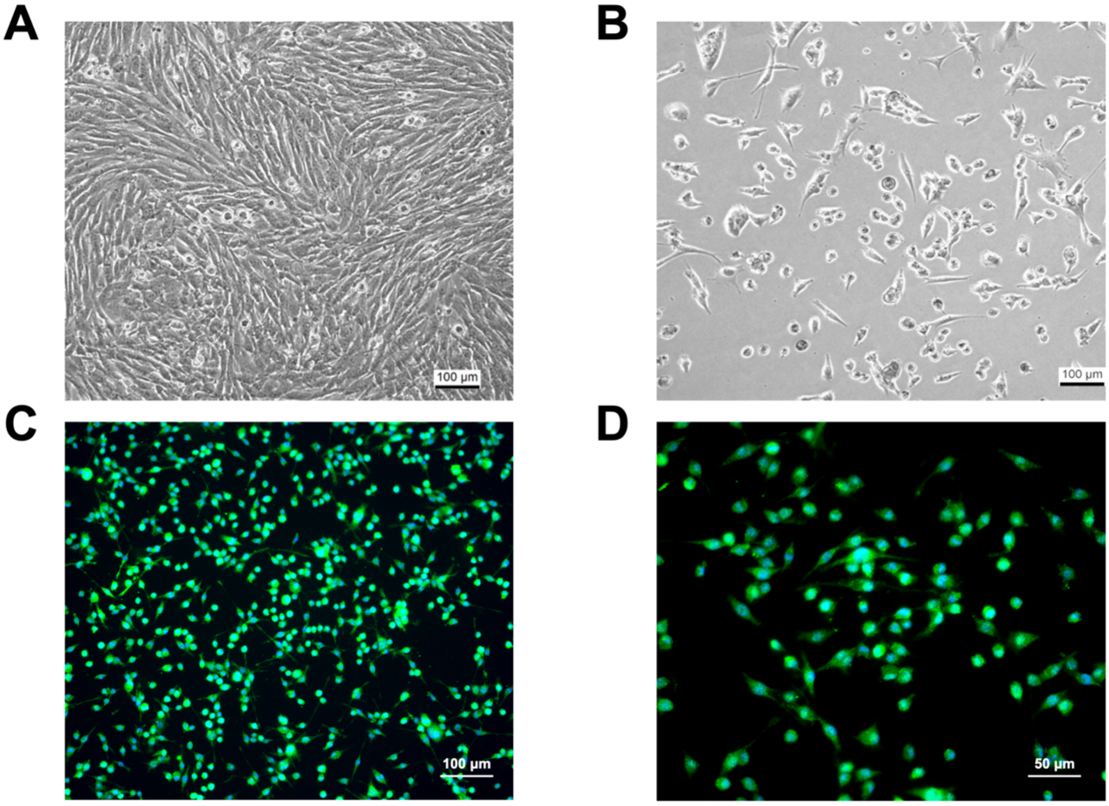

Figure S16. Culture and identification of primary microglia. A Mixed glial cells isolated and cultured from the neonatal mouse cortex. The round cells on the upper layer were naive microglia and oligodendrocyte precursor cells (bar = 100 μm). **B** Representative image of mature microglia replanted for 72 ~ 96 hours after shaking off from mixed cultured glial cells (bar = 100 μm). **C** Representative image of immunostaining. The results of immunostaining showed that more than 95% of cells expressed Iba1 (green), indicating a high purity of primary microglia (bar = 100 μm). **D** Representative image of primary microglia with Iba1 (green) staining under high magnification (bar = 50 μm).

Figure S17.

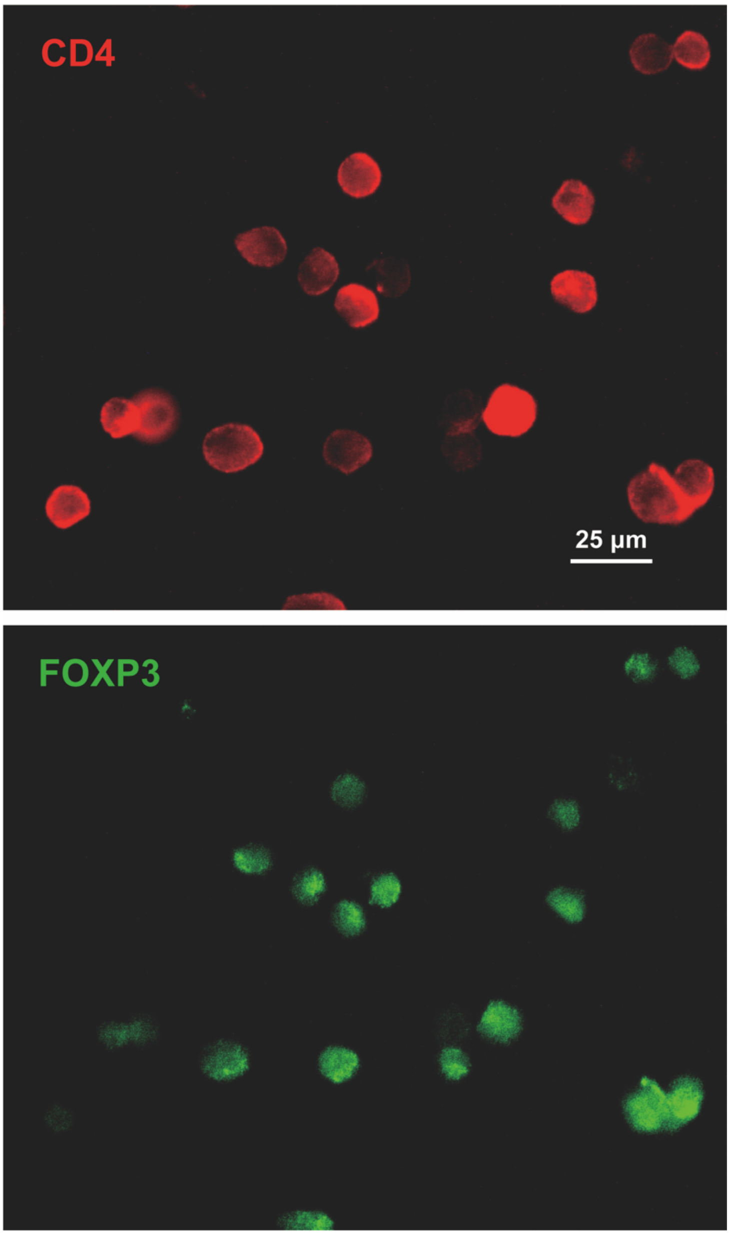

Figure S17. Representative images of immunostaining of Tregs. After sorting by MACS, the cells from the mouse spleen expressed CD4 and FOXP3 simultaneously, which suggested that the cells met the characteristics of Tregs and could be used in subsequent experiments.

Table S1. Hematological data obtained from the tail.

Parameter	Mean ± SD				Normal range	***P value***
	WT	Anti-CD47	SIRPα	SIRPα-v Exo		
Erythrocytes(×10^6/μL)	7.98 ± 0.37	7.27 ± 0.45	7.31 ± 0.71	7.63 ± 0.59	6.5 ~ 10.1	*0.033**
Total White Blood Cells(×10^3/μL)	6.52 ± 1.13	5.31 ± 2.51	5.14 ± 2.26	5.39 ± 2.61	2.6 ~ 11.5	*0.537*
Neutrophils(×10^3/μL)	1.55 ± 0.25	1.93 ± 0.88	1.69 ± 0.27	1.95 ± 0.62	0.4 ~ 2.5	*0.388*
Lymphocytes(×10^3/μL)	2.69 ± 1.22	1.82 ± 1.33	1.55 ± 0.69	1.63 ± 0.91	1.3 ~ 8.4	*0.111*
Monocytes(×10^3/μL)	0.68 ± 0.32	0.56 ± 0.28	0.44 ± 0.17	0.49 ± 0.25	0.3 ~ 1.1	*0.262*
Eosinophils(×10^3/μL)	0.16 ± 0.06	0.13 ± 0.04	0.15 ± 0.05	0.17 ± 0.07	0.065 ~ 0.25	*0.257*
Hemoglobin(g/dL)	12.59 ± 0.79	11.31 ± 1.13	11.19 ± 1.46	12.01 ± 0.84	10.0 ~ 16.1	*0.014**
Mean Corpuscular Volume(fL)	44.17 ± 0.77	46.22 ± 2.25	45.89 ± 2.71	44.71 ± 1.97	42.3 ~ 55.9	*0.133*
Hematocrit(%)	42.06 ± 2.23	37.99 ± 3.67	37.95 ± 3.54	38.56 ± 2.16	32.8 ~ 48	*0.016**
Platelets(×10^6/μL)	915.7 ± 92.5	783.7 ± 78.8	809.9 ± 107.2	868.9 ± 109.1	250 ~ 1540	*0.031**

Blood analysis of C57BL/6 mice. Anti-CD47 antibody and SIRPα variants treatments resulted in a significant decrease in blood cell indices. The results were marked with *. All the data are presented as the mean ± SD. * $P<0.05$

Table S2. Antibodies, concentrations and manufacturers used

Antibodies	Dilutions	Manufacturer	Catalog#
CD29-APC	1:100	eBioscience	17-0291-82
CD44-FITC	1:200	Abcam	ab25064
CD90-FITC	1:250	Abcam	ab25672
CD11b-APC	1:160	CST	41249
CD34-PE	1:500	Abcam	ab223930
CD45-PE	1:300	CST	47742
CD63	1:1000	Abcam	ab217345
CD81	1:1500	Proteintech	66866-1-Ig
Alix	1:1000	CST	92880
MBP	1:5000	Abcam	ab218011
SMI32	1:200	BioLegend	801701
CD16/32	1:200	Abcam	ab223200
CD206	1:200	Abcam	ab64693
Ibal1	1:1000	Wako	013-26471
CD4	1:100	Abcam	ab252152
CD25	1:250	Abcam	ab210332
FOX3	1:200	Abcam	ab215206
phospho-p38 MAPK (Thr180/Tyr182)	1:1000	CST	4511
p38 MAPK	1:1000	Proteintech	14064-1-AP
phospho-STAT1 (Ser727))	1:1000	CST	8826
STAT1	1:1000	Abcam	ab109320
phospho-PI3K (Tyr458)	1:1000	CST	4228
PI3K	1:1000	CST	4257
phospho-Akt (Ser473)	1:1000	Proteintech	66444-1-Ig
Akt	1:1000	CST	4685
phospho-mTOR (Ser2448)	1:1000	CST	2971
mTOR	1:1000	CST	2983
β-actin	1:5000	Sigma	A-5441

Table S3. The sequences of primers used for RT-qPCR

Name	sequences of primers
CD16	(F) TTTGGACACCCAGATGTTTCAG<br>(R) GTCTTCCTTGAGCACCTGGATC
CD32	(F) AATCCTGCCGTTCCTACTGATC<br>(R) GTGTCACCGTGTCTTCCTTGAG
CD86	(F) GACCGTTGTGTGTGTTCTGG<br>(R) GATGAGCATCACAAGGA
CD11b	(F) CCAAGACGATCTCAGCATCA<br>(R) TTCTGGCRRGCTGAATCCTT
CD206	(F) CAAGGAAGGTTGGCATTTGT<br>(R) CCTTTCAGTCCTTTGCAAGC
IL10	(F) CCAAGCCTTATCGGAAATGA<br>(R) TTTTCACAGGGGAGAAATCG
TGF-β	(F) TGCGCTTGCAGAGATTAAAA<br>(R)CGTCAAAAGACAGCCACTCA
YM1/2	(F) CAGGGTAATGAGTGGGTTGG<br>(R) CACGGCACCTCCTAAATTGT
